# Revealing Potential Therapeutic Targets in Gastric Cancer through Inflammation and Protein-Protein Interaction Hub Networks

**DOI:** 10.7150/jca.112218

**Published:** 2025-06-12

**Authors:** Wei Wu, Guoliang Li, Lixin Chai, Yating Yin, Xin Xu, Chenlun Han, Hongyong Liu, Yi Cao, Yumiao Wang, Qunhao Guo, Wenxuan Chen, Peter Wang, Zhijian Pan

**Affiliations:** 1 Department of Gastrointestinal Hepatobiliary Surgery, The Affiliated Hospital of Hangzhou Normal University, Hangzhou City, China.; 2 Department of Medicine, Beijing Zhongwei Research Center, Biological and Translational Medicine, Beijing, 100161, China.

**Keywords:** gastric cancer, inflammation, ScRNA-seq, prognosis, communication

## Abstract

**Background:** Gastric cancer (GC) ranks second in incidence and mortality among digestive system cancer, following colorectal cancer. Currently treatment options are limited, and the prognosis for GC remains poor.

**Methods:** Four bulk RNA sequencing (RNA-seq) datasets and two single-cell RNA sequencing (scRNA-seq) datasets were downloaded from the Gene Expression Omnibus (GEO) database. Initially, we identified differentially expressed genes (DEGs). The intersection list of inflammatory response-related DEGs (IRR-DEGs) was utilized for enrichment analyses. Hub genes were extracted from the protein-protein interaction (PPI) network of DEGs, exploring their expression in the context of scRNA-seq landscapes and cell-cell communication. IRR hub DEGs were identified, and pathway and receptor-ligand pairs were analyzed at this gene level.

**Results:** The analysis identified 69 DEGs in GC. Among these, 8 IRR-DEGs (SPP1, TIMP1, SERPINF1, TNFAIP6, LGALS1, LY6E, MSR1, and SELE) were closely associated with 19 types of immune cells and various lymphocytes. Of the 12 hub genes (SPP1, TIMP1, FSTL1, THY1, COL4A1, FBN1, ASPN, COL10A1, COL5A1, THBS2, LUM, and SPARC), their expression is significantly enhanced in stem cells, primarily involving communication with monocytes, and four prognostic-related genes were discovered. Two IRR hub DEGs indicated that the SPP1 signaling pathway, specifically the SPP1-CD44 ligand-receptor pairs, plays a critical role.

**Conclusion:** We have collectively identified 18 genes that could serve as biomarkers for future GC targeting. The discovery of the SPP1-CD44 ligand-receptor axis not only elucidates a novel inflammatory signaling pathway driving tumor progression, but also provides a potential therapeutic target for disrupting cancer-stromal interactions. Importantly, these biomarkers lay the foundation for developing precision immunotherapies that target the inflammatory-immune axis in GC management.

## Introduction

Gastric cancer (GC) is the fifth most common malignancy worldwide, with the highest age-standardized mortality rate in East Asia at 14.3 per 100,000 individuals 1. The majority of gastric cancers (approximately 90%) are stomach adenocarcinomas (STAD), originating from the epithelial cells in the superficial layer of the gastric wall due to malignant changes in gastric gland cells 2. Although systemic treatments such as radiotherapy, chemotherapy, and surgery have proven effective for GC, a multidisciplinary approach that combines immunotherapy and targeted therapy is essential for comprehensive management 3, 4.

Inflammation plays a crucial role in the development of malignant tumors, as persistent chronic inflammation can impair pathogen clearance and dysregulate innate immunity, thereby facilitating tumorigenesis 5. This mechanism has been extensively studied in pancreatic cancer 6 and colorectal cancer 7 within the digestive system. Notably, the therapeutic potential of targeting inflammation is supported by clinical trials demonstrating that nonsteroidal anti-inflammatory drugs (e.g., aspirin) reduce cancer incidence across multiple tumor types 8, 9. In the context of GC, chronic inflammatory microenvironments not only drive the malignant transformation of precursor lesions 10 but also orchestrate a complex cellular network within the tumor microenvironment (TME). Tumor-associated macrophages, lymphocytes, cancer-associated fibroblasts, and mesenchymal stem cells collectively reinforce inflammation-mediated TME remodeling through reciprocal cytokine signaling, which is a process recently shown to exhibit GC-specific spatial organization patterns 11. This GC-centric inflammatory circuitry highlights the need to identify tumor-stage-specific biomarkers that can distinguish pro-tumorigenic inflammation from physiological immune responses. Therefore, investigating inflammatory biomarkers in the occurrence, progression, and prognosis of GC can provide deeper insights into the underlying mechanisms of cancer.

Since the advent of single-cell RNA sequencing (scRNA-seq) technology combined with bioinformatics 12, scRNA-seq has become a crucial tool for studying the intrinsic mechanisms of cancer cells, as evidenced by numerous published studies 13, 14. In the investigation of intracranial aneurysms, single-cell sequencing has been utilized to elucidate pathophysiology 15 and differentiate transcriptome signatures 16-19. ScRNA-seq aids in gaining insights into the tumor immune microenvironment 19, 20, identifying cell types 21, and predicting cancer survival prognosis 22. Moreover, in non-cancer diseases, such as neuropathic pain, scRNA-seq has been used to explore disease mechanisms and enhance clinical diagnostic knowledge 23, as well as to reveal the heterogeneity and function of valve cells in heart valve diseases 24.

As demonstrated, scRNA-seq has emerged as a powerful tool for dissecting cellular heterogeneity, uncovering developmental trajectories, and resolving disease-associated transcriptional alterations at unprecedented resolution. While previous studies have characterized inflammatory signatures in GC, our work uniquely integrates bulk and single-cell transcriptomics to elucidate the spatial dynamics of inflammation-driven tumor-immune crosstalk. This multi-omics strategy overcomes the limitations of traditional DEG analyses, establishing a paradigm for targeting inflammation-associated cellular niches in GC precision medicine.

## Methods and Materials

### Data collection

All analysis data of this study were sourced from the Gene Expression Omnibus (GEO) database 25. In the selection of the dataset, we prioritized a sample set with balanced data between the tumor and control groups. Ultimately, we downloaded GC transcriptome data (GSE49051, GSE54129, GSE79973, GSE118916) and GC scRNA-seq data (GSE112302, GSE150290 26). The Cancer Genome Atlas (TCGA) database, combined with the Genotype-Tissue Expression (GTEx) database, was used as a validation set to examine the final signature genes obtained.

### Differentially expression analysis

Differentially expressed genes (DEGs) analysis between different groups was performed using the “limma” package in R 27. Data processing screening criteria included a false discovery rate (FDR) < 0.05 and log2 |fold change| ≥ 1 28. The “ggplot2” package was used to create volcano plots showing differential gene expression across the datasets, with the top 10 most significant genes labeled. A Venn diagram was generated using the “VennDiagram” package to visualize the differential genes intersecting across the four datasets, showing the number and percentage of intersecting genes 29.

### Enrichment analysis

After identifying the differentially expressed intersecting genes from the four GC transcriptomes datasets, we used the “org.Hs.eg.db” package to convert gene names and IDs, removing any genes without associated gene IDs. The “clusterProfiler” package 30 was employed to conduct Gene Ontology (GO) 31 and Kyoto Encyclopedia of Genes and Genomes (KEGG) analysis 32 to identify the functions and pathways associated with these genes. The results of both analyses were visualized using bubble and bar plots generated with the “dotplot” function in the “ggplot2” package.

### Identification and analysis of inflammatory response-related genes

Using the Molecular Signature Database (http://gsea-msigdb.org) 33, 34, we derived gene sets of 200 and 850 using “Hallmark Inflammatory Response” and “GOBP Inflammatory Response” as search terms, respectively, resulting in a merged list of 968 genes 35. Inflammatory response-related differently expressed genes (IRR-DEGs) were identified using a Venn diagram. The specific locations of IRR-DEGs on human chromosomes were extracted from annotated documents and visualized using the “RCircos” package 36. The IRR-DEGs list was input into the Transcriptional Regulatory Relationships Unraveled by Sentence-based Text mining (TRRUST, version 2, https://www.grnpedia.org/trrust/) 37 to obtain the transcription factors regulatory network, which was visualized through Cytoscape (Version 3.9.1). The Metascape database (https://metascape.org/) 38 was utilized for the enrichment analysis of IRR-DEGs, including GO biological processes, a network of enriched terms, Cell Type Signatures, and DisGeNET. The GeneMANIA database (http://genemania.org/) 39 was used to analyze gene interactions and functional predictions among IRR-DEGs. The Drug-Gene Interaction database (DGIdb 4.0, http://dgidb.genome.wustl.edu/) 40 provided information on the association of IRR-DEGs with known or potential drugs. To analyze the correlation between inflammation response and immunity, we initially performed immune cell infiltration analysis, retaining results with a P-value less than 0.05. The Spearman method was used to calculate the correlation between 22 types of immune cells and IRR-DEGs, and a heat-map was generated using the “ggplot2” package.

### Protein-protein interaction network and identification of hub genes

The list of intersecting genes was entered into the online tool STRING (Version 12.0, https://cn.string-db.org/) 41, and a minimum required interaction score set to medium confidence (0.400) to obtain the Protein-Protein interaction (PPI) network. Node properties and relationships for drawing the Cytoscape gene network were derived from the PPI network. The network structure was visualized using Cytoscape (Version 3.9.1) 42, with gene node colors and shapes adjusted according to their properties. The top 15 genes with the most connected nodes were extracted using the “cytoHubba” plugin 43, which provides 12 algorithms for calculating hub genes in protein interaction networks. The Maximum Clique Centrality algorithm was selected to identify the hub gene network.

### ScRNA-seq data analysis

Before clustering the screened single-cell sequencing genes, dimensionality reduction was performed to reveal the true structure of the data. Principal component analysis (PCA) was used to select genes representing overall differences, resulting in four principal components 44. The “monocle” package was used to calculate the p-value for each PC. Meanwhile, t-distributed stochastic neighbor embedding (T-SNE) analysis was performed for cell clustering and visualization 45. The “SingleR” package 46 was used to annotate six cell types 47, visualizing the differential expression of 12 selected hub genes in different cell types using violin plots. A scatter plot was used to observe the up-regulated and down-regulated expression trends of these genes, while a bubble plot showed their comparative expression across the six cell types.

### Unsupervised trajectory analysis

Time-series trajectory analysis of cells was performed using the “monocle” package in R 48. The results of the Seurat algorithm were converted into the cell matrix, cell annotation tables, and gene annotation tables needed for the “monocle” package analysis. Cell clustering data was added for cell trajectory analysis, and the final outcomes were annotated into four cell trajectory maps: dendritic, temporal, cell name, and clustering.

### Cell-cell communications analysis

The results of the GC single-cell RNA sequencing analysis were pre-processed using the “cellchat” package 49 and then imported into the “CellChatDB.human” ligand-receptor database for analysis. A pie chart showed the specific composition of the imported database from three perspectives. After pre-processing the single-cell gene expression matrix, the probability of cell-cell communication was calculated, filtering our data with fewer than 10 cells. The calculated findings were summarized and integrated to observe the cell communication status based on the number and intensity of interactions and the individual cell type network graph.

A Venn diagram was used to extract intersecting genes between IRR-DEGs and hub DEGs. Pathways related to inflammatory response hub genes were analyzed by examining ligand-receptor pairs for cell-to-cell communication. Intercellular communication was further inferred at the signaling pathway level and visualized with chord diagrams. Predict cell-cell interactions and cellular action types were analyzed using heat-map observations. Receptor and ligand pairs dominating the hub gene-shared pathways were explored to view the expression levels of interacting genes within the pathway. Cell-cell communication status at the receptor and ligand pair level was displayed with chord diagrams.

### Differential, survival and correlation validation of signature genes

Genes were entered into the GEPIA website (http://gepia.cancer-pku.cn/) 50 to perform differential expression analysis using the “Expression DIY” and to check “Boxplot” for graph shape, color, cancer type and matching database attributes. Validation set data matched TCGA normal and GTEx data to obtain the conclusion on the differential analysis of signature genes. The “Survival” feature was used to generate survival plots for these genes in the prognosis of STAD. Correlation analysis of inflammatory response-associated hub genes was performed using Spearman's rank correlation coefficient to view their correlation with the TPM expression of other signature genes.

### Immune cell-associated expression analysis

To observe whether there was a correlation between lymphocyte content and signature gene expression pairs, signature genes were retrieved from the TISIDB (http://cis.hku.hk/TISIDB/) tool 51. Significant correlations between genes and lymphocyte content were downloaded and saved by selecting the cancer type and lymphocyte name. To explore the correlation between prognosis-related genes and immune cells, the gene names were entered into TIMER 2.0 (http://timer.comp-genomics.org/) 52, and the corresponding immune cell type was selected. STAD was identified in the output heat map, and cells significantly correlated with the genes were selected for graphical output and saving.

### Analysis tools

In this study, the Perl language (Strawberry perl version 5.30.0.1, https://strawberryperl.com/) was used for text processing tasks, such as extracting the GC transcriptome gene expression matrix from the GEO database. For statistical analysis and graph visualization, the R language (R version 4.1.3, https://www.r-project.org/) and its various analysis packages were used.

## Results

### Differential analysis of GC

The GSE49051 database contained 3 normal and 3 tumor samples, identifying 6,674 DEGs. Among the top 10 significantly expressed genes, PRUNE2, C2orf40, and COL4A6 were upregulated, while CFHR2, PAH, CFTR, AGT, SLC13A5, COLEC11, and APOA1 were downregulated (**Figure S1A**). In GSE54129, which included 111 tumor and 21 normal specimens, 1,793 genes were identified as differentially expressed. The expression of TRAPPC1, PCGF1, AP2M1, ERI3, NAPA, PRKCSH, AGO2, Y16709, and VMP1 genes was increased, while VSTM2A, ANKHD1, NCKAP5, PCAT18, EVI5, ENPP6, RP11-108L7.15, ASB8, KCNK10, and AGAP9 expressions were decreased (**Figure S1B**).

In GSE79973, which included 10 normal and 10 tumor tissues, 857 genes showed differential expression. CDH3, SULF1, INHBA, FAM19A5, RAB31, WISP1, COL6A3, ARL13B, and HOXA-AS2 were overexpressed, while CPB1, LOC643201, SULT1B1, PBLD, ZNF57, PIWIL2, GRAMD1C, and CBR1 were under-expressed (**Figure S1C**). GSE118916 had 620 genes with expression discrepancies in 30 types, with CORO1C, CEP170, CD81, C1orf54, GTPBP4, LPCAT1, MSN, RAB31, and FGD6 showing lower expression, and CAPN9, SSTR1, FBP2, LNX1, NRG4, PEX7, CAPN13, and ARSD showing higher expression (**Figure S1D**). A comparison of differential genes across the four datasets yielded 69 intersecting genes, representing 0.9% of all DEGs (**Figure S1E**).

### GO and KEGG enrichment analysis

GO and KEGG enrichment analysis of the 69 DEGs revealed that these genes were primarily associated with the collagen-containing extracellular matrix in the Cellular Component (CC) and with extracellular matrix structural constituent in the Molecular Function (MF) category, showing positive correlations (p < 0.05) (**Figure S2A, B**). In the Biological Process (BP) category, negative correlations were observed in processes such as ossification and wound healing (p < 0.05). Pathway enrichment results indicated that extracellular matrix organization, extracellular structure organization, and external encapsulating structure organization were the most enriched pathways (**Figure S2C, D**).

### Identification and enrichment analysis of IRR-DEGs

**Figure 1:**
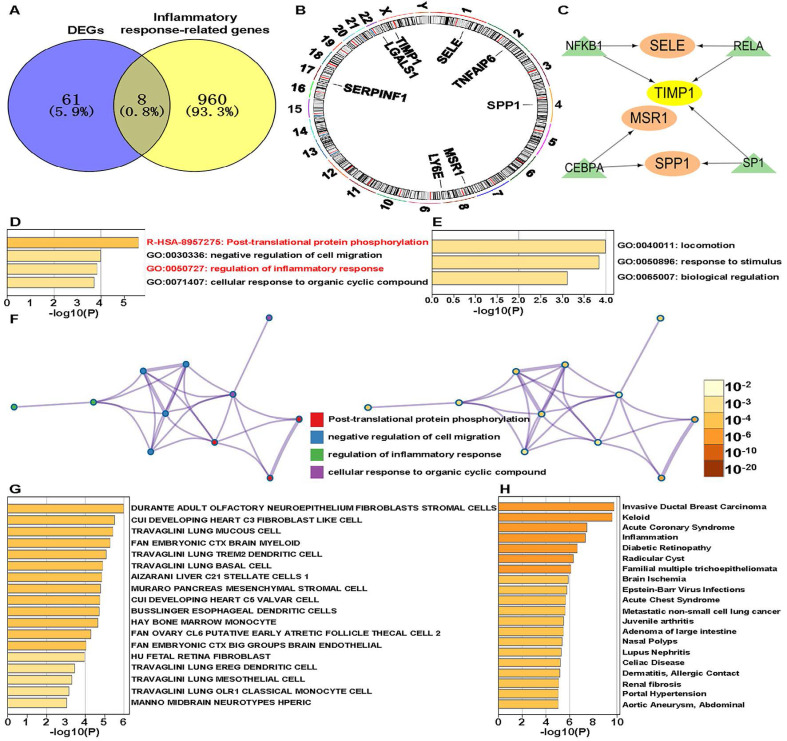
Identification of inflammatory response-related genes. **(A)** Venn diagram showing the intersection of differentially expressed genes (DEGs) and inflammatory response-related (IRR) genes.** (B)** The chromosomal locations of IRR-DEGs across 23 chromosomes.** (C)** Network formed by IRR-DEGs and their transcription factors (TFs). **(D)** Enriched terms of IRR-DEGs, color-coded by p-values.** (E)** The top-level Gene Ontology biological processes.** (F)** Network of enriched terms: the left panel is color-coded by cluster ID and the right panel is color-coded by p-value.** (G)** Summary of enrichment analysis in Cell Type Signatures. **(H)** Summary of enrichment analysis in DisGeNET.

We identified eight IRR-DEGs: SPP1, TIMP1, SERPINF1, TNFAIP6, LGALS1, LY6E, MSR1, and SELE (**Figure 1A**). These genes are located on chromosomes 1, 2, 4, 8, 17, 22, and X (**Figure 1B**). For these, only SPP1, TIMP1, MSR1, and SELE have associated with transcription factors (TFs): TIMP1 is regulated by TFs NFKB1, RELA, and SP1; SELE and SPP1 are regulated by the two regulatory factors, and MSR1 is regulated solely by CEBPA (**Figure 1C**). Enriched terms for these IRR-DEGs included post-translational protein phosphorylation and regulation of the inflammatory response (**Figure 1D**). In the GO biological process function, genes involved in locomotion were most enriched (**Figure 1E**). The network diagram illustrated notable enrichment in the negative regulation of cell migration and cellular response to organic cyclic compounds (**Figure 1F**). Significant enrichment was also observed in adult olfactory neuroepithelium fibroblasts and stromal cells in the Cell Type Signatures analysis. Enrichment analysis using the DisGeNET database revealed a strong association with Invasive Ductal Breast Carcinoma and Keloid (**Figure 1G**).

### Constructing PPI and DGI networks for IRR-DEGs

**Figure 2:**
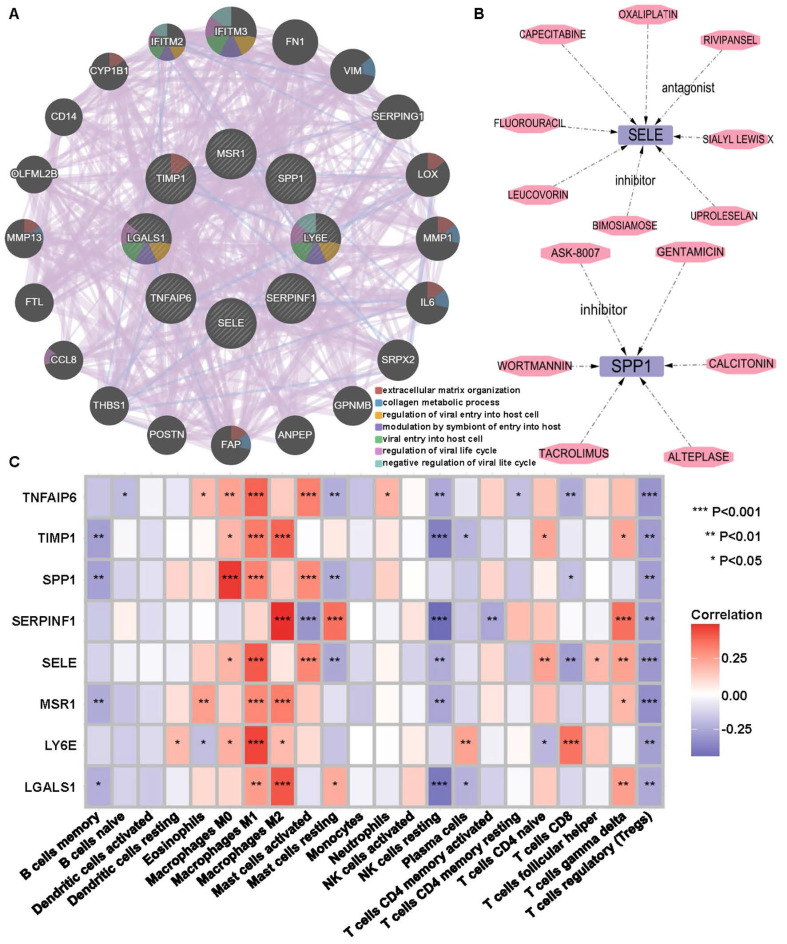
Inflammatory response-related DEGs (IRR-DEGs) protein-protein interaction (PPI) network, drug-gene (DGI) interaction network and immune association analyses. **(A)** PPI network for 8 (IRR-DEGs based on GeneMANIA database.** (B)** DGI network, with SELE on the top and SPP1 on the bottom.** (C)** Correlation of 22 immune cells with IRR-DEGs. *p < 0.05, **p < 0.01, and ***p < 0.001.

The PPI network revealed interactions between the 8 IRR-DEGs and 20 other genes, primarily focusing on extracellular matrix organization, collagen metabolic process, and regulation of viral entry into host cells (**Figure 2A**). In the Drug-Gene Interaction (DGI) Network, 8 drugs were found to target SELE and 6 drugs targeted SPP1. Rivipansel and Biomosiamose are antagonists for SELE, while ASK-8007 is an inhibitor for SPP1 (**Figure 2B**).

### Association of IRR-DEGs with immunity and lymphocytes

The 8 IRR-DEGs exhibited significant correlations with 19 immune cells, with T regulatory cells (Tregs), resting NK cells, and memory B cells showing negative correlations, and M0, M1, and M2 macrophages showing positive correlations (p < 0.05) (**Figure 2C**).To investigate the impact of cancer inflammatory response-related genes on lymphocytes, the Spearman algorithm was used to analyze the correlation of the 8 IRR-DEGs with 28 lymphocytes using the TISIDB database, which includes data from 415 STAD patients. The top 4 lymphocyte types with the highest correlation coefficients for SPP1 were Treg, Tγδ, Tcm_CD8, and activated dendritic cells (Act_DC), showing the strongest positive correlations (p <0.001) (**Figure S3A-P, S4A-P**). TIMP1, SERPINF1, TNFAIP6, LGALS1, and SELE were also predominantly positively correlated with lymphocytes. Among the top 4 lymphocyte types correlated with LY6E, eosinophil and Tem_CD4 showed negative correlations (p < 0.05). Notably, MSR1 exhibited strong correlations with lymphocytes, with multiple rho values exceeding 0.7 (p < 0.05). The 8 IRR-DEGs were analyzed for differential expression between different subgroups based on data from 408 tumor and 211 normal samples from TCGA and GTEx (**Figure S5A-H**). Six IRR-DEGs showed significant differences between subgroups (p < 0.05), with only SERPINF and SELE not statistically significant. In survival analysis, SERPINF1 and SELE were associated with patient prognosis, while other genes showed less favorable prognosis (**Figure S5I, P**).

### Protein interaction network and hub genes analysis

**Figure 3:**
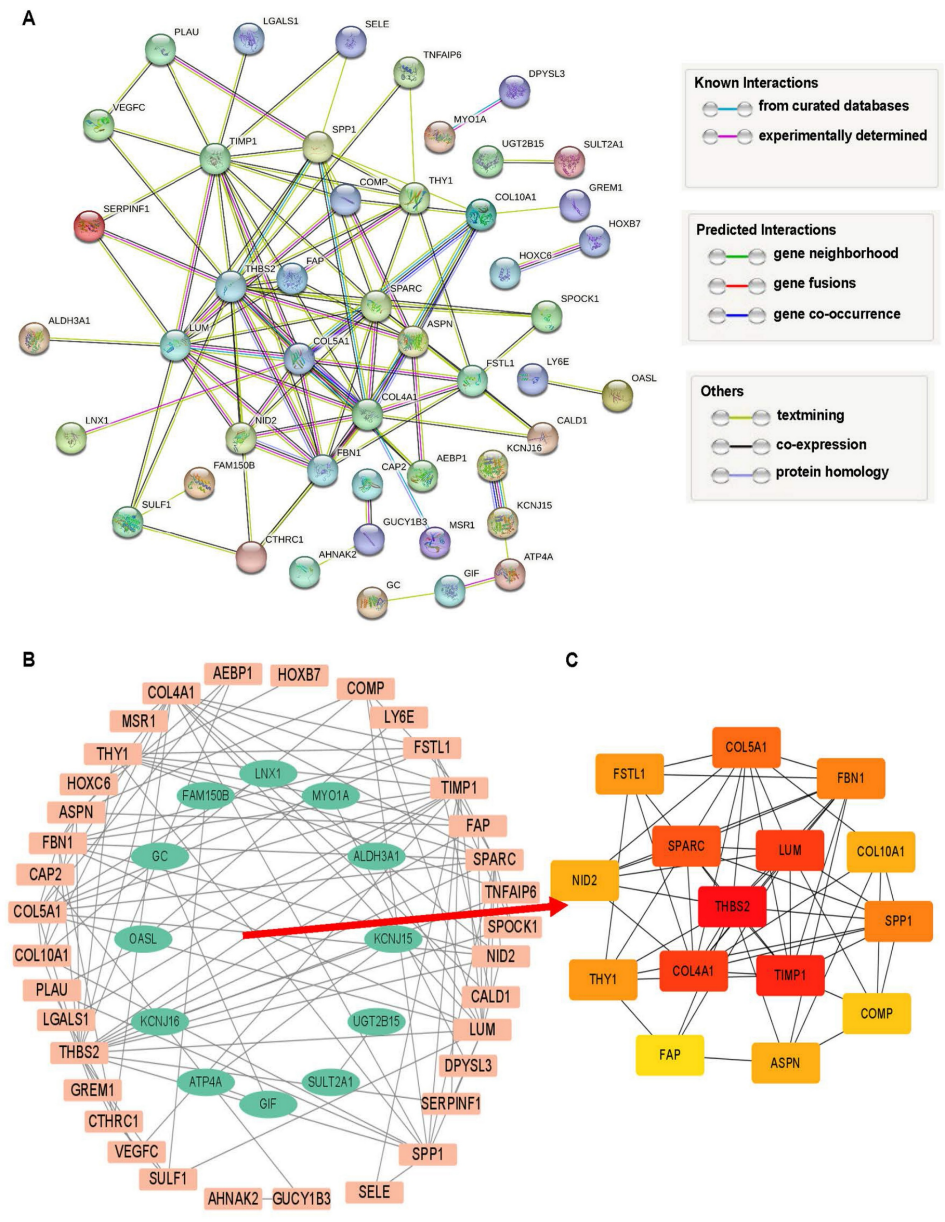
Analysis of intersection DEGs. **(A)** PPI network of 69 DEGs relied on STRING database.** (B)** Interaction network of upregulated (orange) and downregulated (green) genes.** (C)** The top 15 hub DEGs within the interworking network.

The 69 DEGs were subjected to PPI analysis, revealing protein interactions between genes. Nodes represent genes, connecting lines represent interactions, and line colors indicate different evidence (**Figure 3A**). PPI results were exported and imported into Cytoscape, where downregulated genes were represented by green nodes and upregulated genes by orange nodes, with connecting lines indicating interactions (**Figure 3B**). Fifteen hub network genes were identified using the “cytoHubba” plugin, with THBS2, SPARC, LUM, COL4A1, and TIMP1highighted as important center-hub genes due to their high connectivity (**Figure 3C**).

### Quality control and filtering of ScRNA-seq data

The single-cell GSE150290 database of GC includes 29 normal and 23 tumor samples, and GSE112302 has 10 specimens (6 tumors). The number of genes per sample varied (**Figure S6A**). In GSM3067368, GSM3067369, GSM3067370, GSM3067373, GSM3067374, and GSM3067375, the number of sequenced genes in six samples exceeded 6,000, while the number of genes in the rest of the samples was less than 4,000. Sequencing depth for these six samples exceeded 750,000 (**Figure S6B**). The mitochondrial content was zero for all samples (**Figure S6C**). No correlation was found between sequencing depth and mitochondrial gene content (**Figure S6D**), but a strong correlation was observed with gene number (R=0.64) (**Figure S6E**). The intercellular coefficient of variation was calculated, extracting the top 3,000 genes with the largest variation among 8,611 genes for subsequent analysis. APOE, GKN1, TYROBP, TAGLN, LIPF, GAST, and MUC6 genes had the largest fluctuation coefficients (**Figure S6F**).

### ScRNA-seq data PCA downscaling and TSNE clustering

Four PCA principal components were set, visualizing the 20 feature genes of each principal component using bubble plots (**Figure S7A-D**). PC_1 and PC_3 showed both positive and negative expression, while PC_2 showed only negative and PC_4 only positive expressions. PCA heat maps indicated high (yellow) and low (purple) expression levels, highlighting the top 5 significantly expressed genes for each principal component (**Figure S7E-H**). The 29 sample cells were downscaled, with each color representing one sample (**Figure S7I**). All 20 PCA components were included in subsequent analysis (**Figure S7J**). T-Distributed Stochastic Neighbor Embedding (T-SNE) clustering showed that all cells were clustered into 16 cell clusters (**Figure S7K**). The cells were annotated into six cell types: epithelial cells, endothelial cells, tissue stem cells, smooth muscle cells, B cells and monocyte (**Figure S7L**).

### Distributed expression of hub genes on ScRNA-seq data

**Figure 4:**
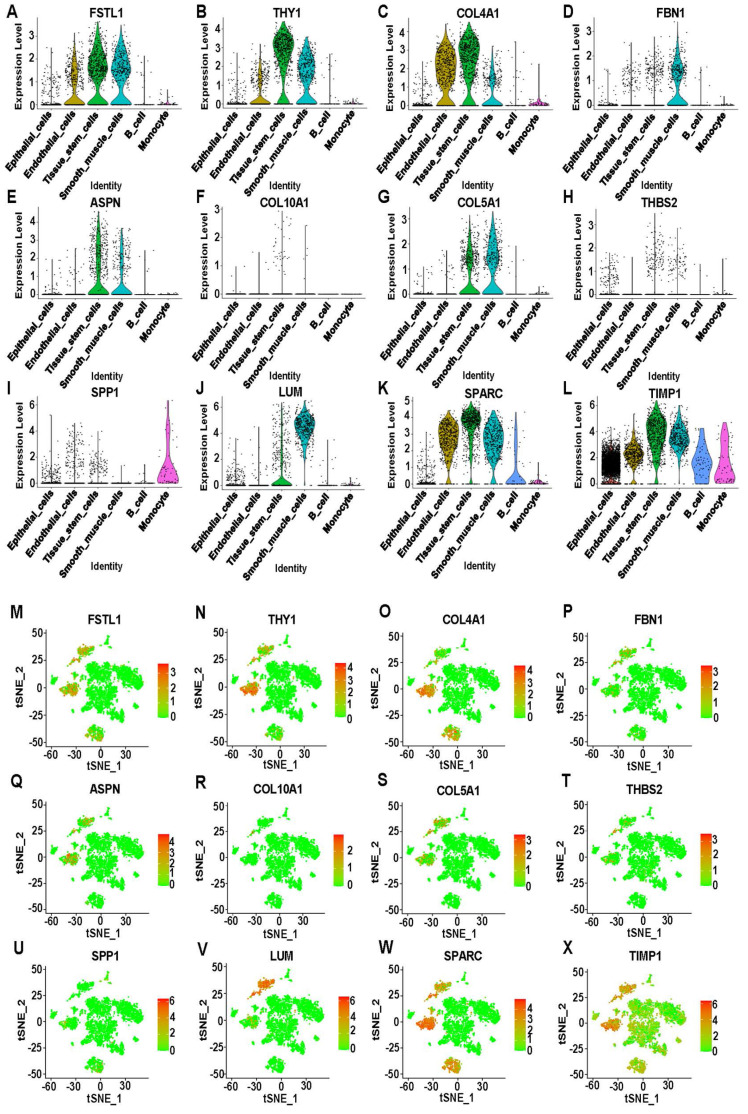
Expression analysis of 12 hub DEGs on scRNA-seq data. **(A-L)** Violin plots showing the differential expression of 12 hub DEGs across cell types.** (M-X)** T-Distributed Stochastic Neighbor Embedding (T-SNE) plots showing the expression of 12 hub DEGs in sixteen clusters. Red indicates upregulation, and green indicates downregulation.

Twelve genes were selected from the 15 hub genes: FSTL1, THY1, COL4A1, FBN1, ASPN, COL10A1, COL5A1, THBS2, SPP1, LUM, SPARC, and TIMP1. Violin plots showed that FSTL1, THY1, and COL4A1 were highly expressed in endothelial cells, tissue stem cells, and smooth muscle cells. Interestingly, COL10A1 and THBS2 were lowly expressed in all cells. TIMP1 was the only gene that was enhanced in all cells, while the remaining genes were mostly overexpressed in tissue stem cells and smooth muscle cells (**Figure 4A-L**). Moreover, the t-SNE scatter plot of hub genes against cell type annotation showed upregulated status in cells, with redder dots indicating higher gene expression (**Figure 4M-X, S7L**). A bubble plot lists all genes in a coordinate system to compare expression differences in different cell types (**Figure S8A**).

### Unsupervised trajectory analysis of cells state transitions

Trajectory analysis and the cell state analysis graph showed three unused states in the process of cell differentiation (**Figure S8B**). Combined with pseudo time analysis, the darker blue color of branch 1 represented the earlier division time, indicating that cell differentiation started from branch 1, then moved to branch 2, and finally merged into branch 3 (**Figure S8C**). Six annotated cell types indicated that cells started to differentiate from epithelial cells (**Figure S8D**). Differences in cell trajectories were observed by examining different cell clusters (**Figure S8E**).

### Identification of prognosis-related hub gene signatures in STAD

The expression of the 12 hub genes showed consistent with statistical significance between TCGA and GTEx groups (p < 0.05) (**Figure S9A-L**). Data from 192 STAD patients showed that ASPN, COL4A1, FSTL1, and NID2 genes were associated with patient survival, with higher survival rates in low-risk patients (**Figure S9M-P**). Using the TIMER 2.0 method, three types of immune cells, including CD8+ T cell, macrophage, and neutrophil, were significantly associated with prognosis-related differentially expressed genes (PR-DEGs). Scatter plots reflected the relationship between PR-DEGs expression and immune cell content, showing a negative connection with purity and a positive one with these three immune cells (**Figure S10A-L**).

The association between PR-DEGs and lymphocyte content was validated, and five statistically significant lymphocytes were selected for visualization: Mast cells, NK cells, NKT cells, Act_CD4 T cells, and CD56dim cells. Scatter plots showed positive correlation with Mast cells, NK cells and NKT cells, and negative trends for Act_CD4 T cells and CD56dim cells. The ASPN, COL4A1, and FSTL1 genes showed negative correlation with lymphocytes, while NID2 showed positive correlation with these lymphocytes (**Figure S11A-T**).

### Cell-cell communications between main six cell types

Ligand-receptor pairs in the cell communication database were divided into three types: secreted signaling (61.8%), extracellular matrix-receptor (21.7%) and cell-cell contact genes (16.5%). The main interactions consist of others (52.1%) and heterodimers (47.9%), with 73% of ligands and receptors originating from KEGG and 27% from Literature (**Figure S12B**). Network diagrams of cell-cell communication interactions showed nodes representing cell types, with larger nodes indicating a greater number of cells (**Figure S12C**). For example, epithelial cells (green) acted as ligand cells sending signals to monocyte (recipient cells). The strength of cell-cell communication interactions was indicated by connecting lines (**Figure S12D**). The network graph for individual cell types described the state of cell-cell communication with other cell types (**Figure S12E-J**).

### Signaling pathway analysis base on cell-cell communication

**Figure 5:**
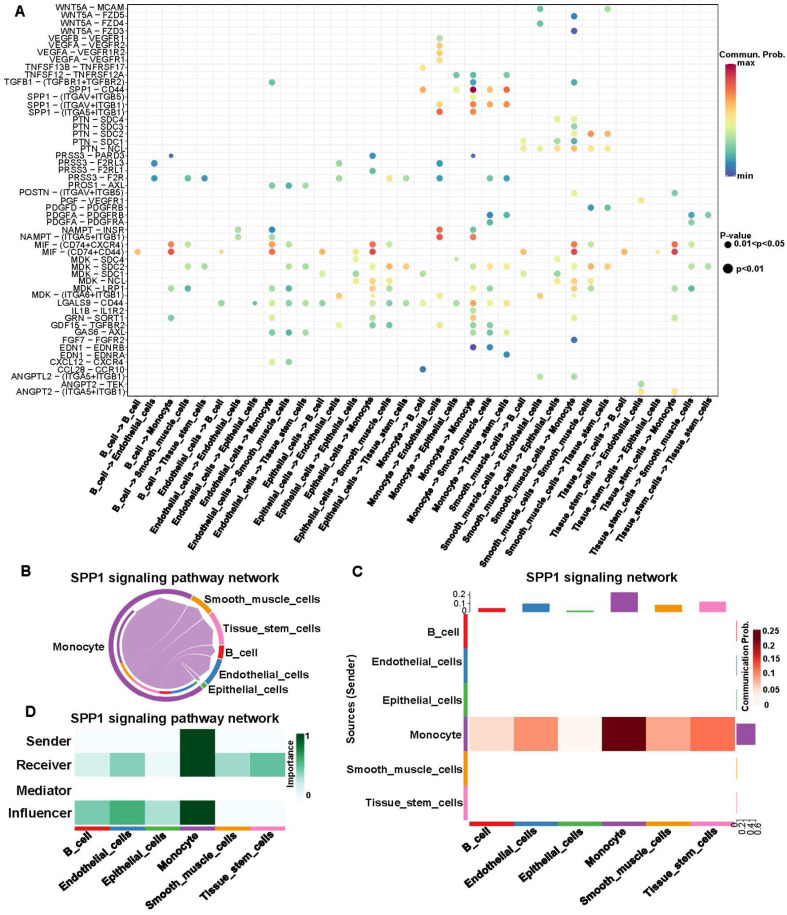
Signaling pathway analysis for cell-cell communication of inflammation-related hub genes. **(A)** Expression of ligand-receptor pairs in interacting cells.** (B)** Cell-cell communication based on the SPP1 signaling pathway.** (C)** Heatmap of cell-cell communication within the SPP1 signaling pathway. The red color indicates the likelihood of cellular interactions.** (D)** Types of cell Interactions within the SPP1 signaling pathway.

IRR-DEGs and hub DEGs intersected at SPP1 and TIMP1 (**Figure S12A**). It is worth mentioning that in the bubble plot, SPP1-CD44 had the smallest P-value for monocyte self-interactions. In interactions with other cells, SPP1-(ITGAV+ITGB5), SPP1-(ITGAV+ITGB1), and SPP1-(ITGA5+ITGB1) played significant roles (**Figure 5A**). Monocytes primarily acted as ligand cells sending signals to other cells, which served as receptor cells (**Figure 5B**). The heat-map showed that monocyte-monocyte interactions were most probable (**Figure 5C**). Monocytes acted as both sender and receiver in the SPP1 signaling pathway network, with the largest information flow (**Figure 5D**).

**Figure 6:**
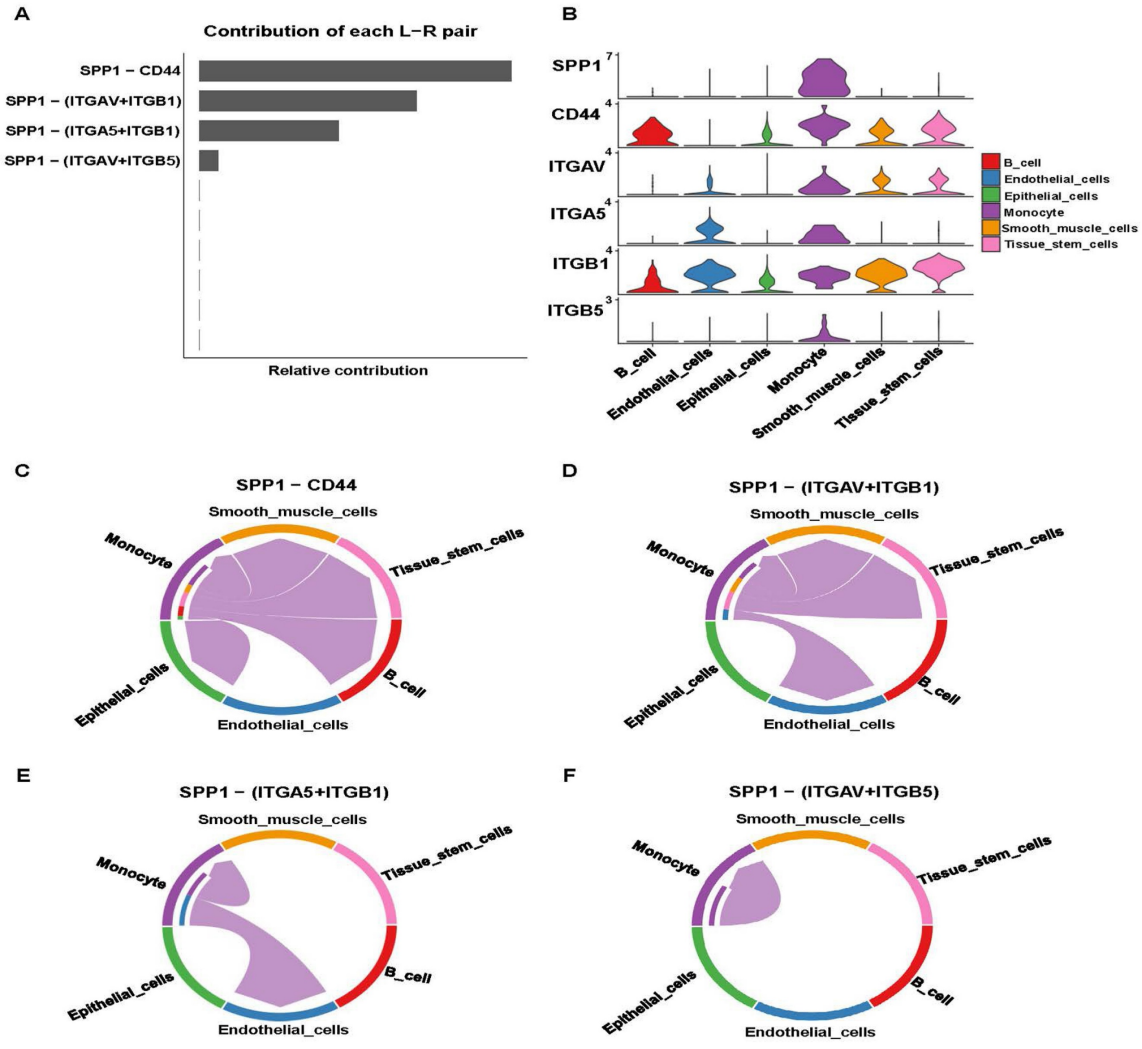
Analysis of the SPP1 signaling pathway. **(A)** Identification of effective ligand-receptor pairs in the SPP1 signaling pathway.** (B)** Expression levels of reciprocal genes in the SPP1 signaling pathway.** (C-F)** Cell-Cell communications of four effective ligand-receptor pairs.

SSP1-CD44 played the most significant role in cell-cell communication, followed by SPP1-(ITGAV+ITGB1), SPP1-(ITGA5+ITGB1), and SPP1-(ITGAV+ITGB5) (**Figure 6A**). Pathway genes were differentially expressed in cell types, with all genes overexpressed in monocyte. ITGAV, CD44, ITGB1 genes were highly expressed in smooth muscle cells and tissue stem cells (**Figure 6B**). Cell-cell communication at the ligand-receptor pair level of the SPP1 signaling pathway showed monocyte did not interact with endothelial cells in the SPP1-CD4 group. In SPP1-(ITGAV+ITGB1), monocyte did not interact with epithelial cells and B cells. Monocytes interacted only with themselves and endothelial cells in SPP1-(ITGA5+ITGB1). In SPP1-(ITGAV+ITGB5), monocyte did not interact with other cells (**Figure 6C-F**).

### Expression relevance of inflammatory response-related hub DEGs

**Figure 7:**
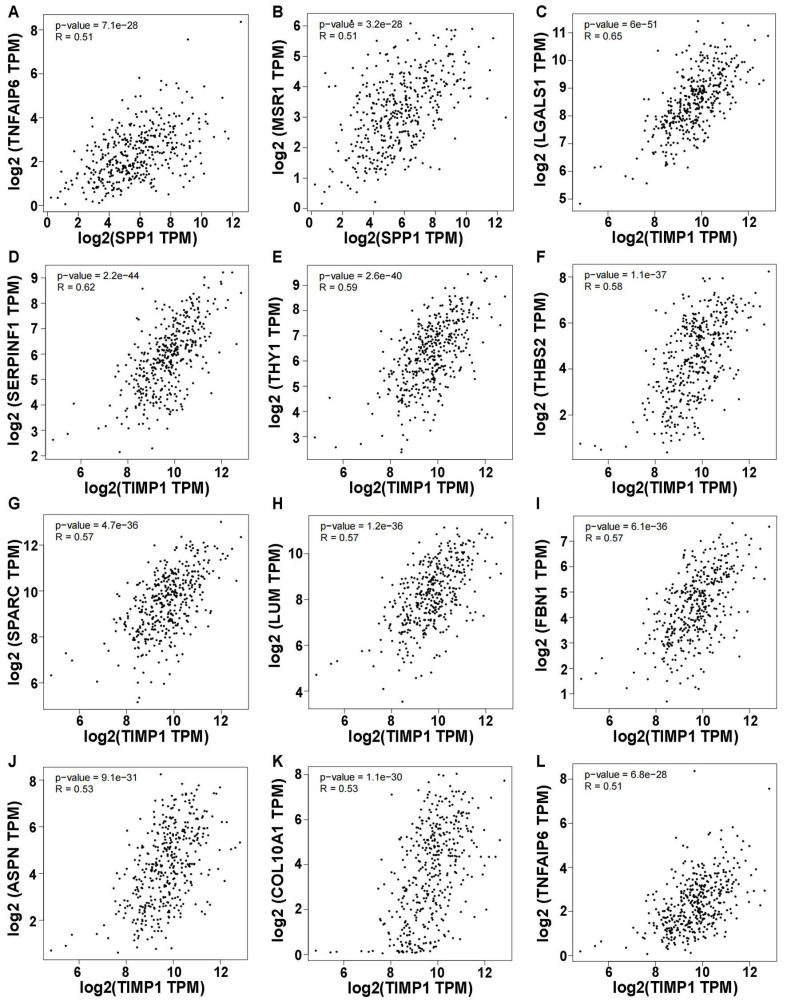
Correlation of SPP1 and TIMP1 with other gene expression (Only correlation coefficients greater than 0.5 are shown and p value < 0.05). **(A)** SPP1-TNFAIP6. **(B)** SPP1-MSR1. **(C)** TIMP1-LGALS1. **(D)** TIMP1-SERPINF1.** (E)** TIMP1-THY1. **(F)** TIMP1-THBS2. **(G)** TIMP1-SPARC. **(H)** TIMP1-LUM. **(I)** TIMP1-FBN1. **(J)** TIMP1-ASPN. **(K)** TIMP1-COL10A1. **(L)** TIMP1-TNFAIP6.

Using a correlation coefficient exceeding 0.5, genes showing elevated self-expression alongside SPP1 expression included TNFAIP6 and MSR1 (p < 0.05) (**Figure 7A, B**). Ten genes exhibited heightened expression levels with variations in TIMP1, namely LGALS1, SERPINF1, THY1, THBS2, SPARC, LUM, FBN1, ASPN, COL10A1, and TNFAIP6 (**Figure 7C-L**). Other genes had correlation coefficients below 0.5 (**Figure S13A-V**). Intriguingly, all genes demonstrated positive correlations with SPP1 and TIMP1, warranting future research.

## Discussion

In the current study, the first section explores the characteristics of eight inflammation-related factors in GC, including chromosomal location, transcription factors, enriched terms, GO analysis, Cell Type Signatures, disease enrichment analysis, PPI networks, DGI networks, immune cells, and lymphocytes. The functions, enriched pathways, potential drug targets, and effects on immunity and lymphocytes of inflammatory 8 IRR-DEGs in GC are analyzed from multiple perspectives. The differential expression and prognostic value of these biomarkers are validated using external datasets. In the second section, the Maximum Clique Centrality algorithm is employed to identify 12 hub genes from PPI networks. Through scRNA-seq data and pseudotime analysis of cellular states, the status of these12 hub DEGs at the cellular level is examined, with a focus on differences, survival, and correlations with immunity and lymphocytes. SPP1 and TIMP1 are identified as both inflammation-related and hub genes. Cell-cell communication analysis reveals cell associations and the contribution of receptor-ligand pairs based on SPP1. The Spearman method is used to calculate their correlation with the expression levels of other genes.

The primary etiology of GC is believed to be chronic infection by Helicobacter pylori, leading to an active inflammatory microenvironment 53. After surgical resection of advanced GC, systemic inflammatory parameters indicate a significant increase in absolute lymphocyte count among survivors, suggesting that enhanced immune function and an increased systemic inflammatory response may impact prognosis 54. The analysis of 8 IRR-DEGs has revealed enriched terms related to the regulation of inflammatory response and post-translational protein phosphorylation. Protein translational modifications (PTMs) contribute to processes such as DNA repair, immune response, metabolism, histone regulation, and kinase regulation, with phosphorylation playing a role in DNA repair imbalance 55. Interleukins play a crucial role in cancer, and their rational use can improve the effectiveness of immunotherapy while limiting side effects 56. The analysis has shown that SPP1 can upregulate the expression of interferon-γ and interleukin-12, while TNFAIP6 can be induced by pro-inflammatory cytokines such as TNF-α and interleukin-1. These findings contribute to a deeper understanding of the mechanistic role of inflammation in GC, thereby providing valuable insights for GC diagnosis and treatment.

The key finding of our study is the identification of SPP1 and TIMP1 as common factors in both the IRR-DEGs and hub DEGs lists. TIMP1 is highly expressed in scRNA-seq data, with the highest distribution in epithelial cells. Additionally, the miR-6745-TIMP1 axis has been shown to suppress cell growth and metastasis in GC 57. Prospective cohort studies indicate that higher levels of TIMP1 in patients with GC and colorectal cancer are associated with poorer prognosis 58. SPP1 is noteworthy as it is exclusively expressed in monocytes. Monocyte-derived cells can modulate the immunotherapeutic response and, consequently, influence the efficacy of cancer therapy 59. Key monocyte features may serve as potential therapeutic targets 60. Additionally, the SPP1-CD44 ligand-receptor pair in the SPP1 signaling pathway plays a prominent role in cell-cell communication.

Targeting immune cells represent one of the most promising therapeutic strategies in oncology. Antibodies against programmed cell death protein 1 (PD-1) and its ligand PD-L1 are among the most frequently employed immunotherapeutic agents and have achieved some success in treating GC 61. However, meta-analyses indicated that PD-1/PD-L1 inhibitors have not reached a valuable threshold in GC treatment 62. Emerging approaches, including tumor vaccines, nanotechnology, have advanced personalized and optimized immunotherapy for GC patients 63. Research has demonstrated that various cytokines and their receptors can enhance the anti-tumor capacity of chimeric antigen receptor (CAR) T-cell therapy 64. Our analysis revealed a significant correlation between multiple inflammation-related factors and various T- cell populations. Additionally, nanomaterial-based modulation of tumor-associated macrophages has been proven to be a viable approach for treating digestive system tumors 65. Most of the inflammation-related factors we discovered exhibited a significant positive correlation with macrophages. Other immune cells, including NK cells, mast cells, B cells, and eosinophils, were also associated with inflammatory factors, highlighting their potential as novel targets for GC immunotherapy.

The hub genes ASPN, COL4A1, FSTL1, and NID2 are associated with prognostic survival in STAD patients. ASPN has two distinct effects on GC cells: HIF1α-mediated resistance to oxidative stress via glucose metabolism reprogramming, and activation of CD44-Rac1 and MMP9 to promote GC cell migration and invasion 66. COL4A1 is associated with gastric cancer peritoneal metastasis through weighted gene co-expression network analysis and clinical specimen validation 67. Other researchers corroborate our findings, asserting that COL4A1 plays a pivotal role in the etiology, diagnosis, and prognosis of GC 68, 69. Mechanistic studies show that FSTL1 promotes proliferation, migration, and invasion in GC, partially by activating AKT via regulation of TLR4/CD14 70. FSTL1 knockdown may promote cell apoptosis via the STAT6 signaling pathway 71. One study demonstrates that upregulated NID2 plays an important role in promoting the invasion and migration of GC cells, serving as a potential biomarker for diagnosis 72.

TGF-β1 has been reported to play a vital role in the development of various diseases, including cancer 73, 74. Antagonists of TGF-β1 can reverse the oncogenic effects attributed to the heightened expression of LGALS1 75. Conversely, inhibiting THBS2 expression is linked to the promotion of epithelial-mesenchymal transition (EMT) in GC 76. Meta-analysis reveals a close association between overexpression of SPARC and diminished survival rates among GC patients 77. SPARC enhances the chemosensitivity of GC cell lines to 5-Fluorouracil (5-FU) through EMT inhibition 78. FBN1 may correlate with drug resistance in GC 79, present in the form of K672-succinylated modifications 80. The TGF-β1-SOX9 axis-inducible COL10A1 fosters invasion and metastasis in GC via EMT transition 81. Elevated expression of TNFAIP6 exacerbates the invasive capacity of GC cells 82, 83. NAT10 fosters GC metastasis through N4-acetylated COL5A1 84. MSR1 orchestrates the GC progression by promoting M2 macrophage polarization 85. Silencing of COL4A1 suppressed the malignant progression of GC 86. Diminished expression of LY6E augments cancer cell apoptosis 87. SERPINF1 has been implicated in immunoregulation associated with vascular mimicry in GC, yet its precise mechanistic role remains elusive 88. The expression of LUM holds pivotal prognostic significance for GC 89-91, thereby making it a beacon for future experimental inquiry. Mechanistic insights into the role of SELE in GC remain undeveloped.

We revealed cell-type-specific expression patterns (e.g., SPP1 in monocytes) and ligand-receptor interactions (e.g., SPP1-CD44), offering novel insights into GC heterogeneity and therapeutic targeting. Notably, the dual roles of SPP1 and TIMP1 as both inflammatory mediators and hub genes underscore their potential as combinatorial therapeutic targets. However, certain limitations should be considered. First, reliance on public datasets may introduce batch effects or population bias. Second, single-cell annotations depend on existing marker genes, potentially overlooking rare subpopulations. Third, bioinformatic predictions require experimental validation (e.g., RT-qPCR, Western blotting, or immunohistochemistry). To address these challenges, future studies should prioritize: 1) multi-center cohorts integrating proteomics and spatial transcriptomics to enhance generalizability. 2) Machine learning-augmented cell annotation combined with flow sorting for refined subpopulation characterization. 3) Functional validation using patient-derived organoids or cell lines to confirm therapeutic potential. This work establishes the foundation for precision oncology in GC by linking inflammatory dynamics to actionable targets. Translational efforts should focus on validating these candidates in preclinical models and clinical trials, ultimately advancing personalized diagnostic and immunotherapeutic strategies for GC patients.

## Supplementary Material

Supplementary figures.
