# Supplementary material for: Revealing Potential Therapeutic Targets in Gastric Cancer through Inflammation and Protein-Protein Interaction Hub Networks
Source: J Cancer. 2025 Jun 12;16(8):2720–36. doi: 10.7150/jca.112218 (PMC12170991; doi:10.7150/jca.112218)
Supplement: Supplementary file 1 — Supplementary figures. [file jcav16p2720s1.pdf]

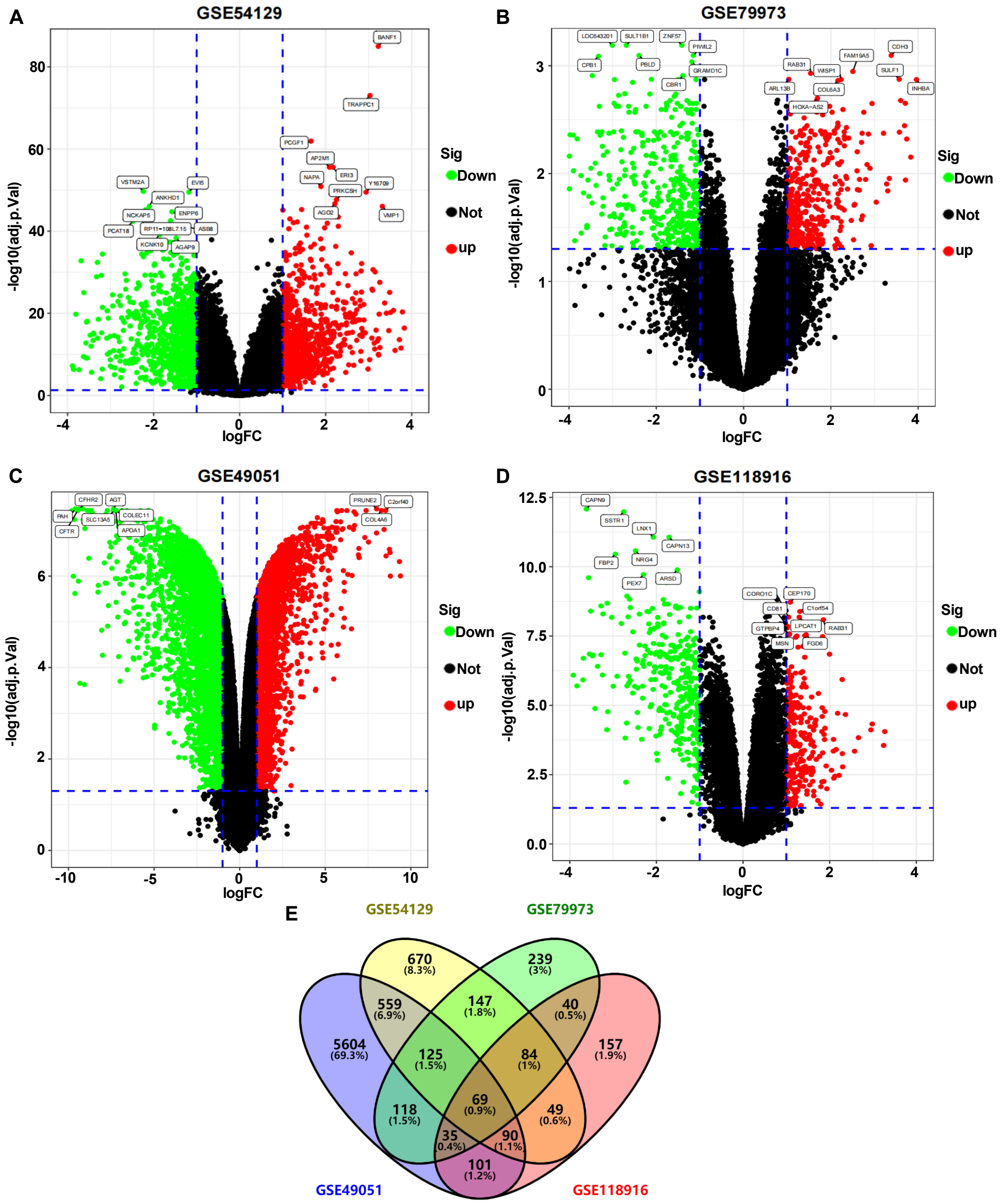

Figure S1: Differential gene expression analysis of GEO gastric cancer (GC) data.

(A) Differentially expressed genes in GSE54129.

(B) Differentially expressed genes in GSE79973.

(C) Differentially expressed genes in GSE49051.

(D) Differentially expressed genes in GSE118916.

(E) Venn diagram showing the intersecting genes across four GEO database.

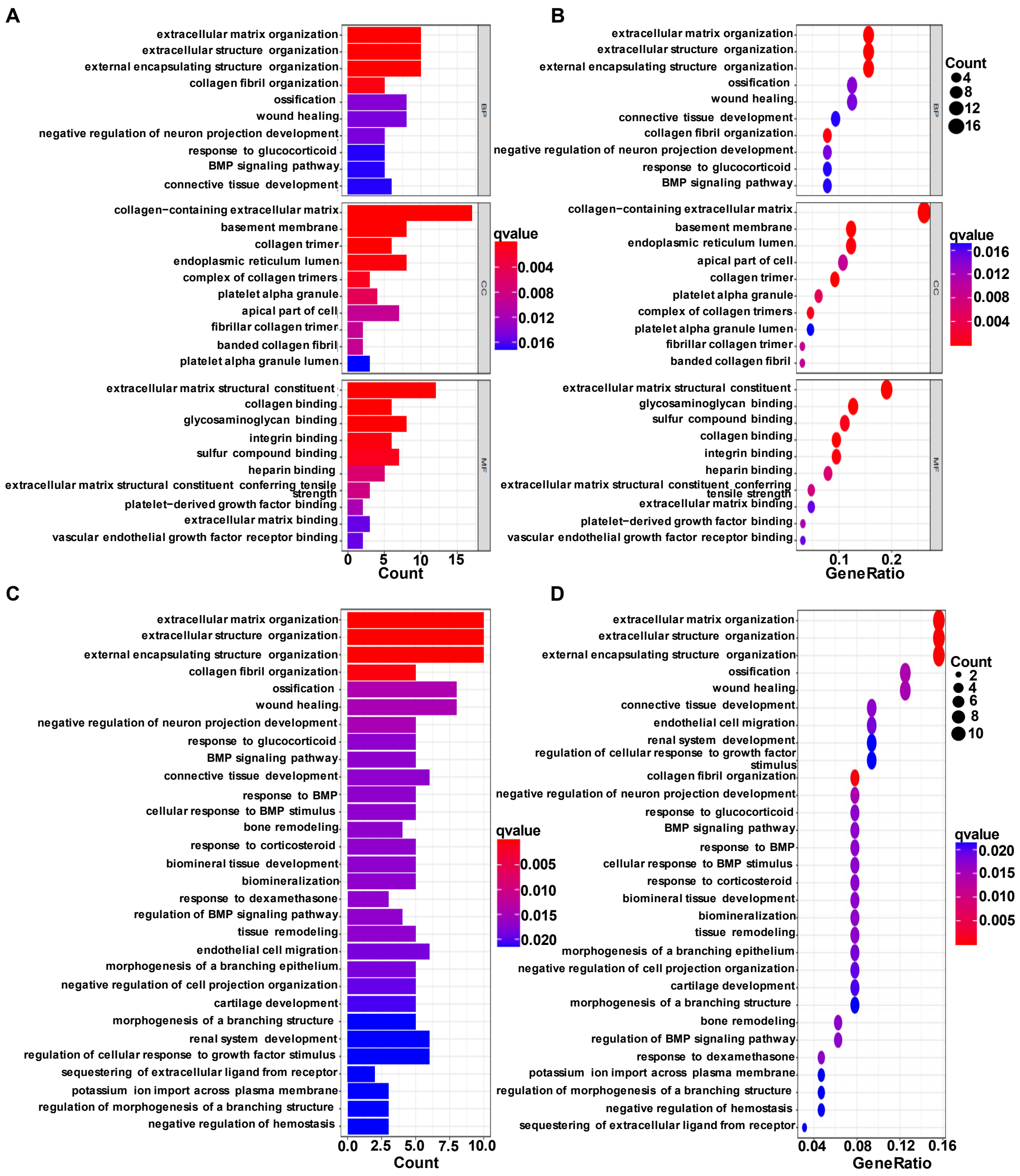

Figure S2: Enrichment analysis of 69 intersecting differently expressed genes (DEGs).

(A) Bar plot of GO analysis.

(B) Bubble chart of GO analysis.

(C) Bar plot of KEGG analysis.

(D) Bubble chart of KEGG analysis.

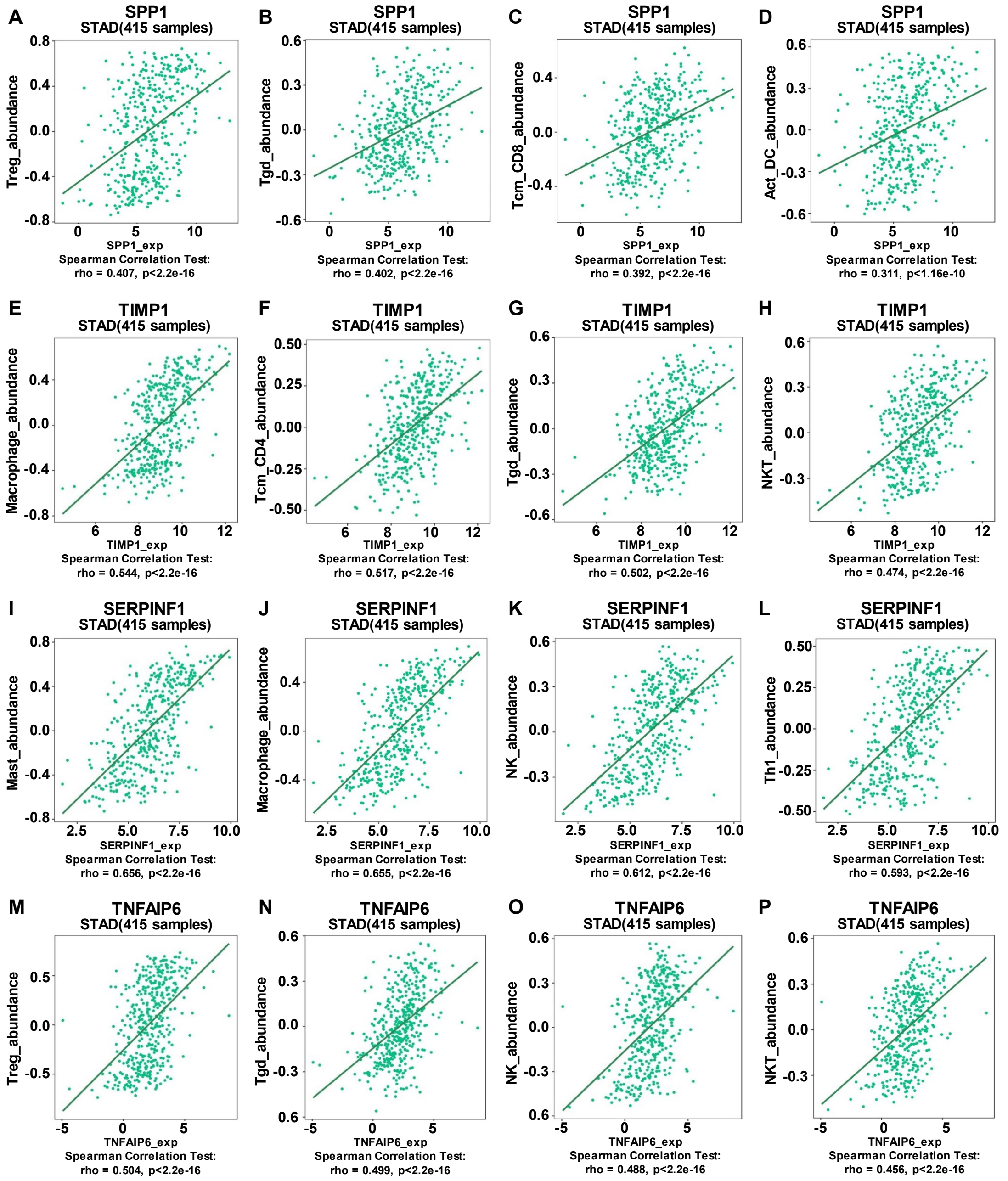

Figure S3: Correlation of SPP1, TIMP1, SERPINF1, and TNFAIP6 with lymphocytes (Top 4 with the max correlation coefficient shown). **(A)** SPP1-Regulatory T cell (Treg). **(B)** SPP1-Gamma delta T cell (Tγδ). **(C)** SPP1-Central memory CD8 T cell (Tcm_CD8). **(D)** SPP1-Activated dendritic cell (Act_DC). **(E)** TIMP1-Macrophage. **(F)** TIMP1-Central memory CD4 T cell (Tcm_CD4). **(G)** TIMP1-Tγδ. **(H)** TIMP1-Natural killer T cell (NKT). **(I)** SERPINF1-Mast cell (Mast). **(J)** SERPINF1-Macrophage. **(K)** SERPINF1-Natural killer cell (NK). **(L)** SERPINF1-Type 1 T helper cell (Th1). **(M)** TNFAIP6-Treg. **(N)** TNFAIP6-Tγδ. **(O)** TNFAIP6-NK. **(P)** TNFAIP6-NKT.

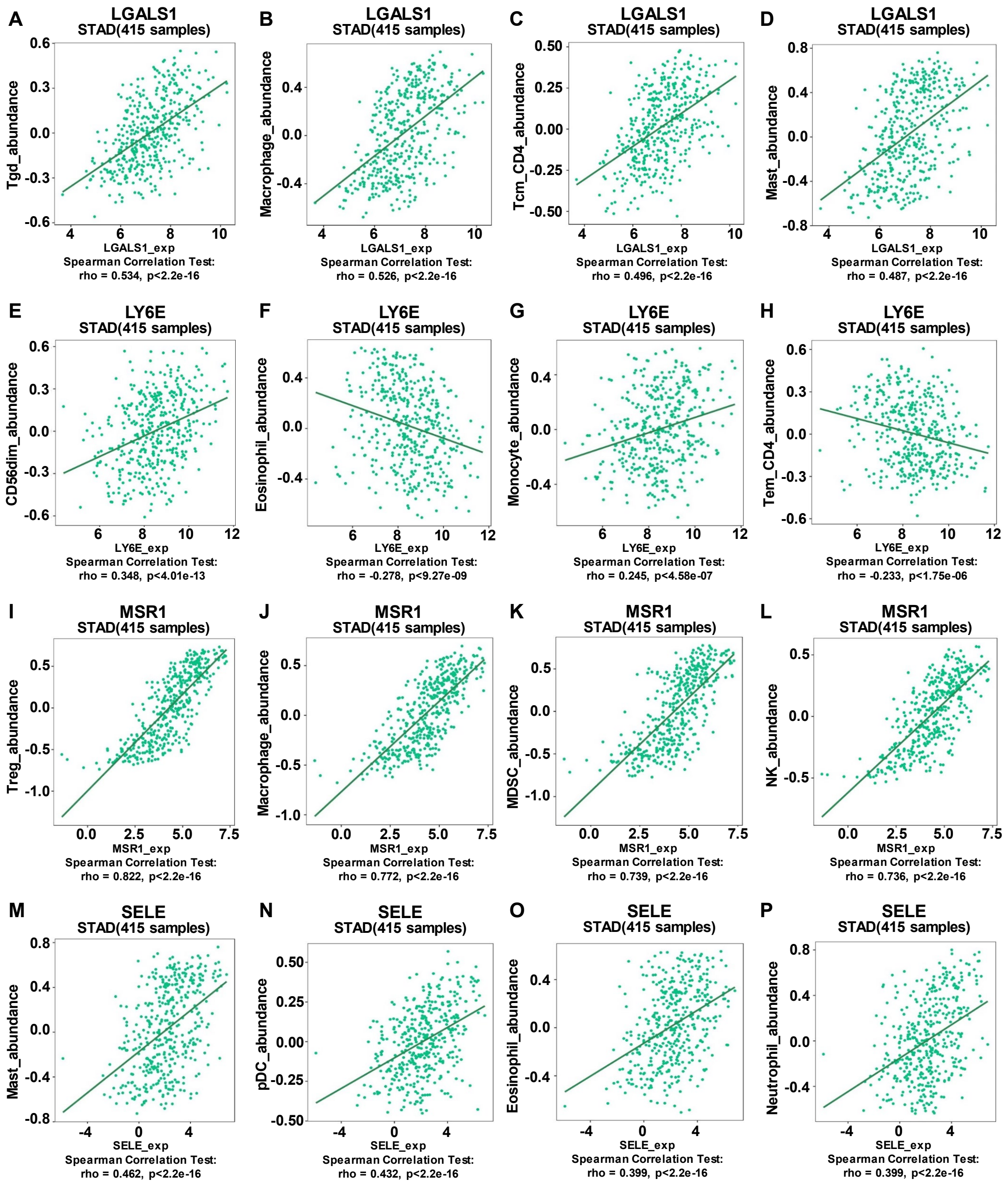

Figure S4: Correlation of LGALS1, LY6E, MSR1, and SELE with lymphocytes (Top 4 with the max correlation coefficient shown). **(A)** LGALS1-Gamma delta T cell (Tγδ). **(B)** LGALS1-Macrophage. **(C)** LGALS1-Central memory CD4 T cell (Tcm_CD4). **(D)** LGALS1-Mast cell (Mast). **(E)** LY6E-CD56dim natural killer cell (CD56dim). **(F)** LY6E-Eosinophil. **(G)** LY6E-Monocyte. **(H)** LGALS1-Effector memory CD4 T cell (Tem_CD4). **(I)** MSR1-Regulatory T cell (Treg). **(J)** MSR1-Macrophage. **(K)** MSR1-Myeloid derived suppressor cell (MDSC). **(L)** MSR1-Natural killer cell (NK). **(M)** SELE-Mast cell (Mast). **(N)** SELE-Plasmacytoid dendritic cell (pDC). **(O)** SELE-Eosinophil. **(P)** SELE-Neutrophil.

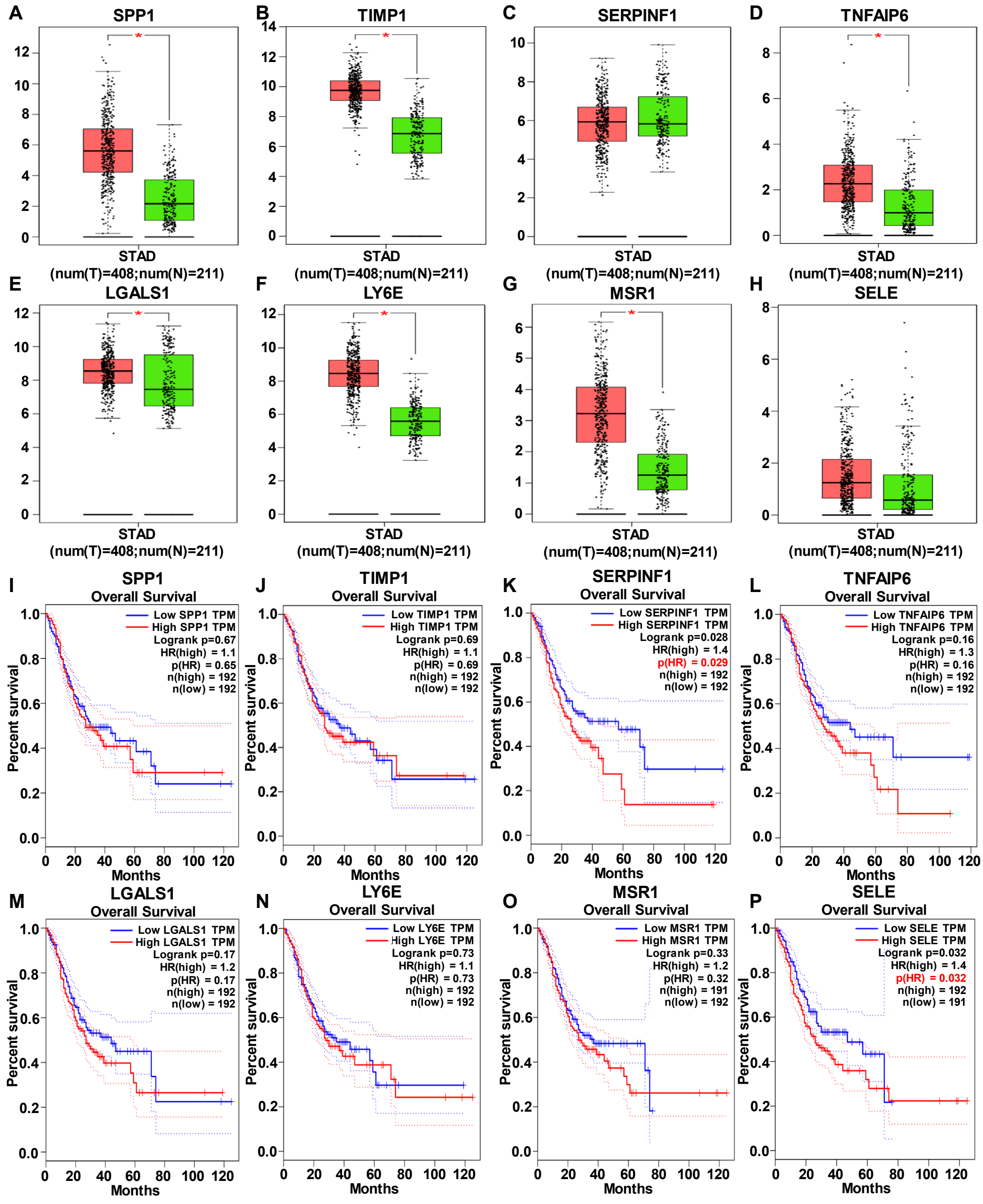

Figure S5: Differential expression and survival analyses for validation of 8 IRR-DEGs.

(A-H) Differential analysis of 8 IRR-DEGs matched TCGA normal and GTEx data (* P < 0.05).

(I-P) Identification of progression associated with STAD prognosis.

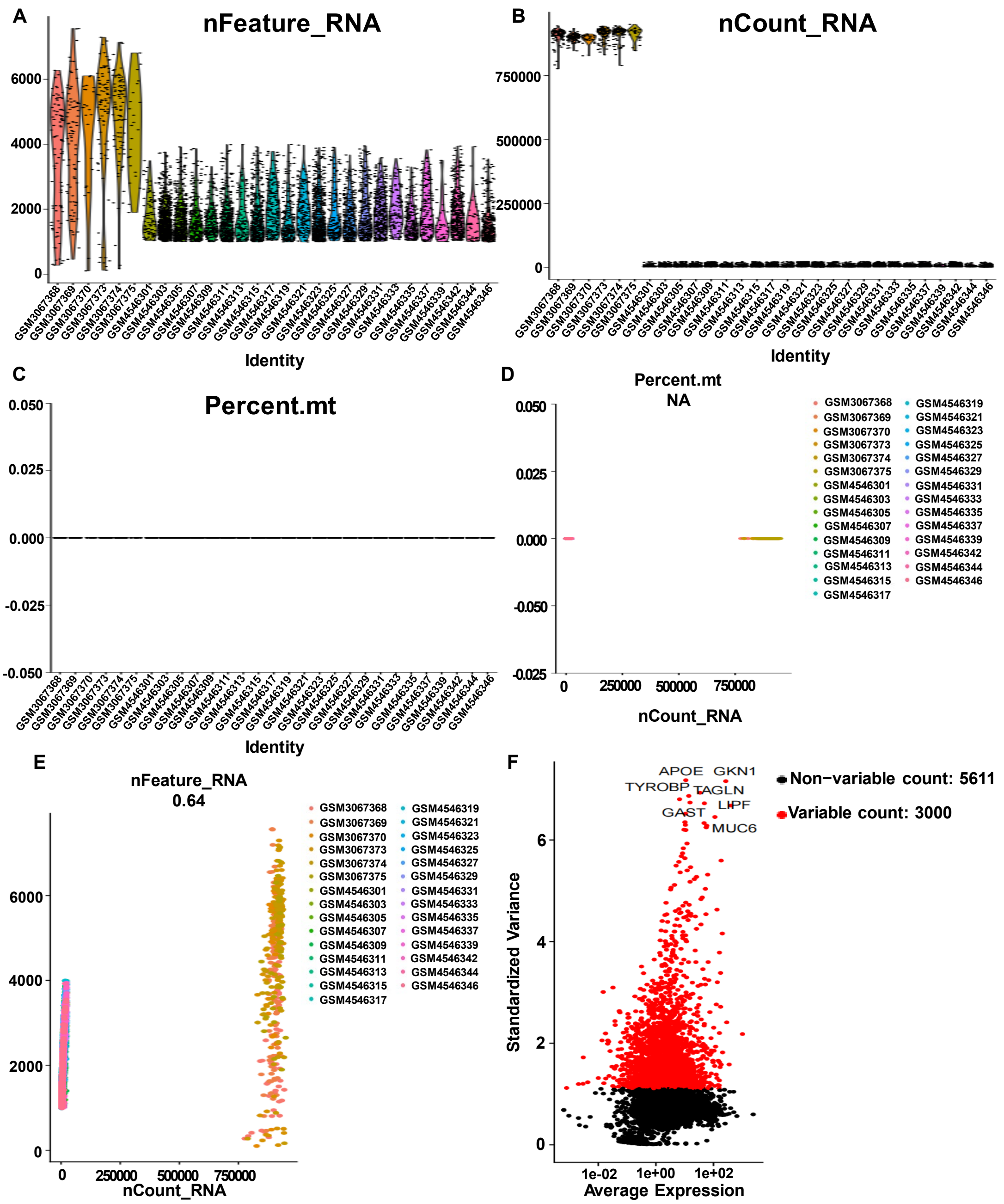

Figure S6: Quality control and filtering of scRNA-seq data.

(A) Number of genes detected in 29 GC samples. **(B)** Sequencing depth in each sample.

(C) Content of mitochondria genes in each sample. High levels indicate low cell activity.

(D) Relationship between sequencing depth and mitochondrial gene content.

(E) Positive correlation between sequencing depth and total intracellular sequences (R=0.64).

(F) Volcano plot illustrating genes fluctuating in all samples. The top 3,000 genes marked in red had high variations, and the names of the top 10 genes are presented.

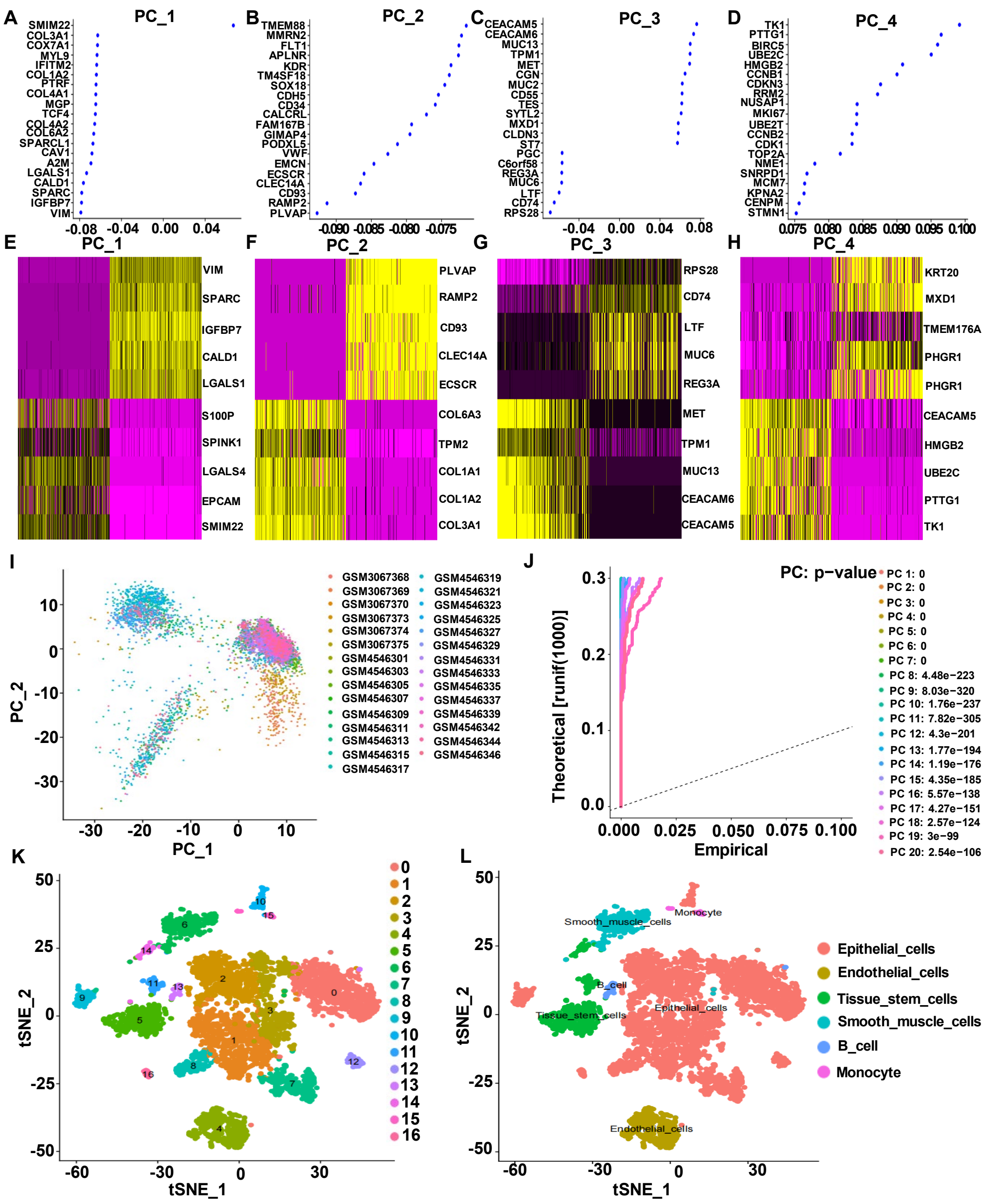

Figure S7: ScRNA-seq data PCA downscaling and tSNE clustering.

(A-D) Bubble diagram of marker genes in each PCA cluster (top 20). **(E-H)** Heatmap of marker genes in each PCA cluster. Yellow represents upregulated genes, and purple indicates downregulated genes (top 5). **(I)** PCA of scRNA-seq data for preliminary dimensionality reduction. **(J)** The p-values of each PCA were displayed, and the smallest 20 were selected for subsequent analysis. **(K)** T-SNE clustering of GC cells. **(L)** Cell type annotation of clusters. Sixteen clusters were annotated as epithelial cells, endothelial cells, tissue stem cells, smooth muscle cells, B cells, and monocyte.

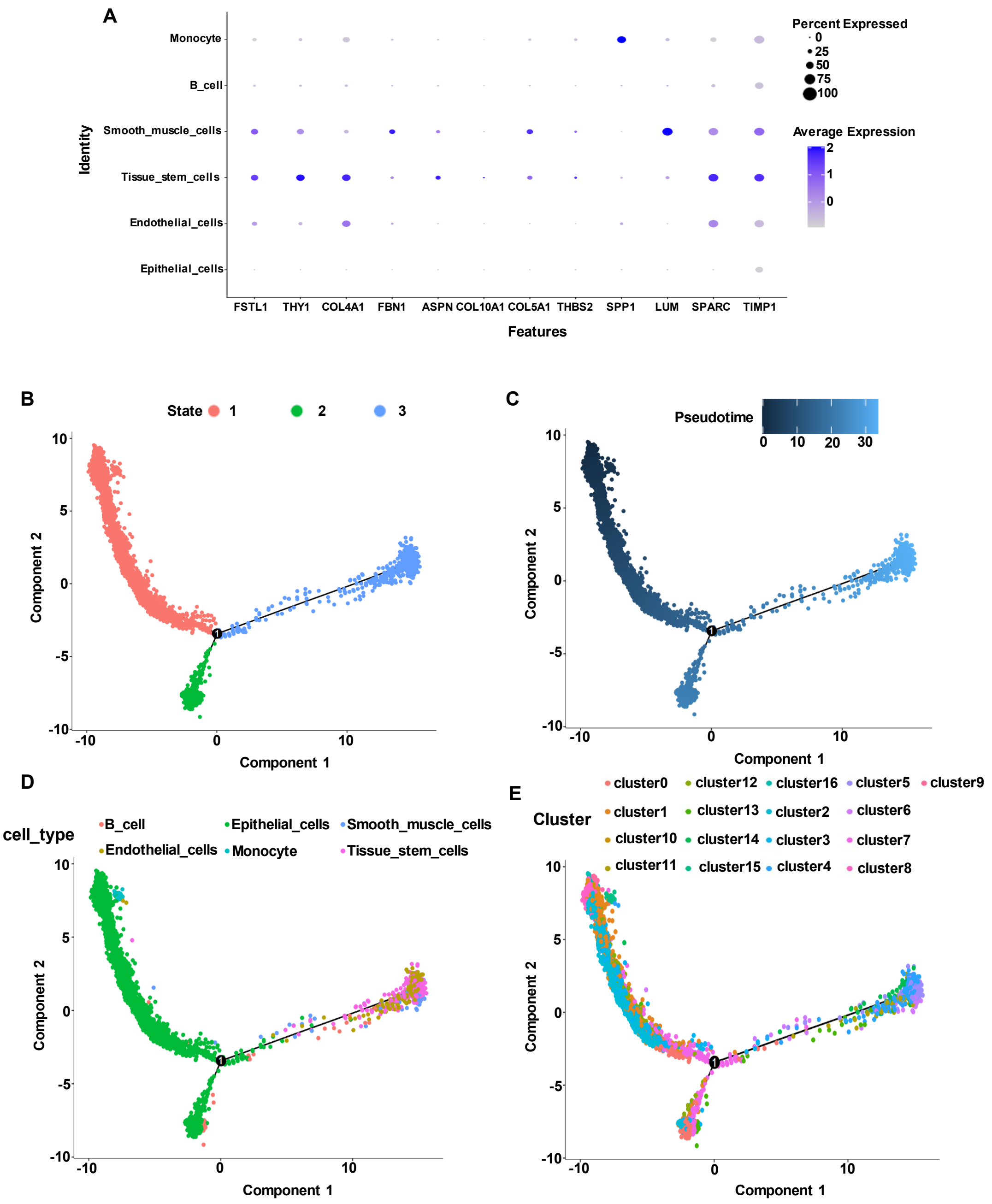

Figure S8: Analysis of intersecting gene interaction.

(A) Bubble map showing the expression of 12 hub genes in different cell types. Darker blue color indicates higher gene expression.

(B-E) Unsupervised trajectory of monocyte state transitions in GC samples. The branched trajectory was colored by cell states, pseudo-time, cell types, and cell clusters.

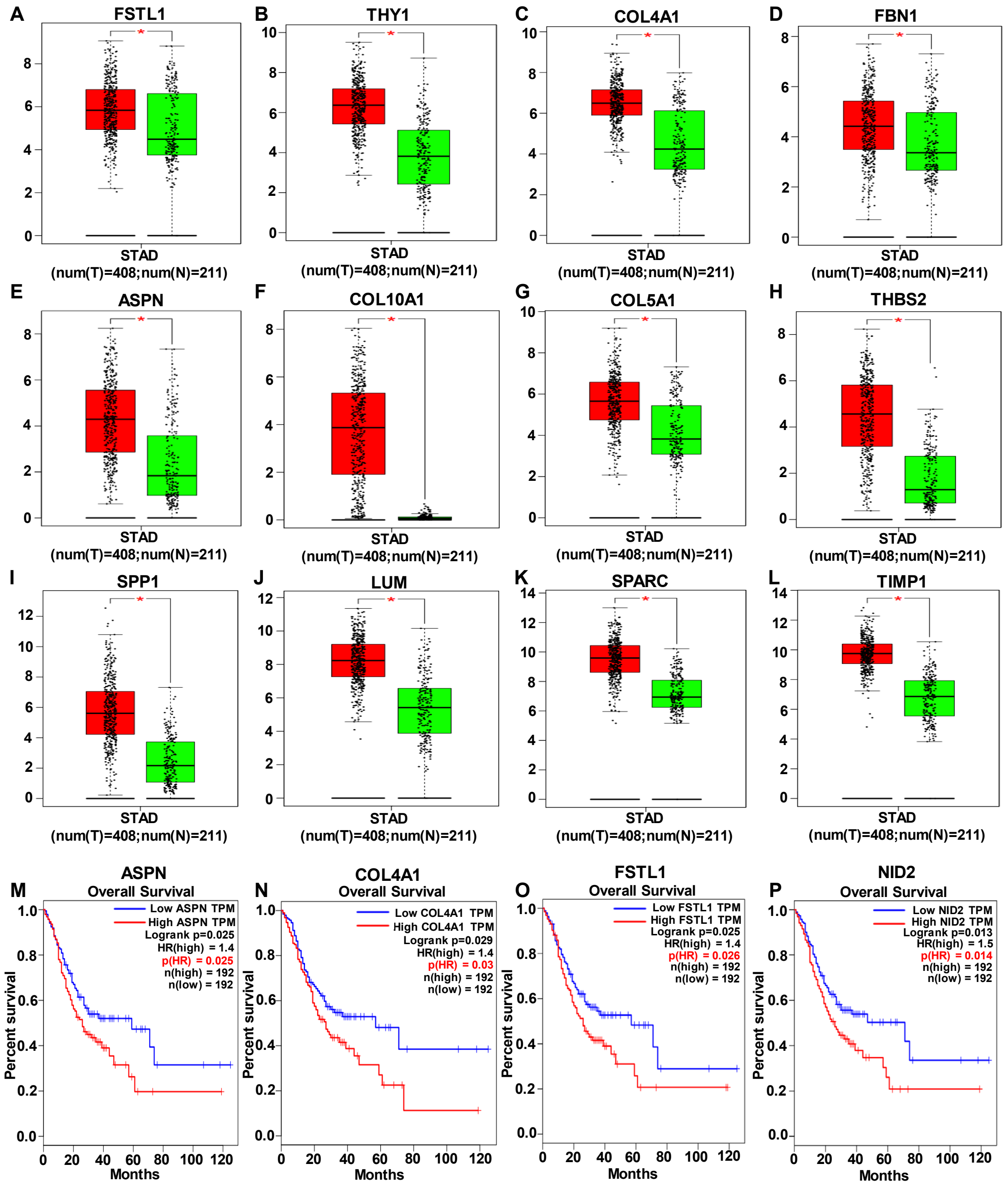

Figure S9: Identification of prognosis-related gene signatures in STAD.

(A-L) Differential analysis of 12 hub DEGs matched with TCGA normal and GTEx data (* P < 0.05).

(M-P) Survival analysis of prognosis-related genes among the 12 hub genes.

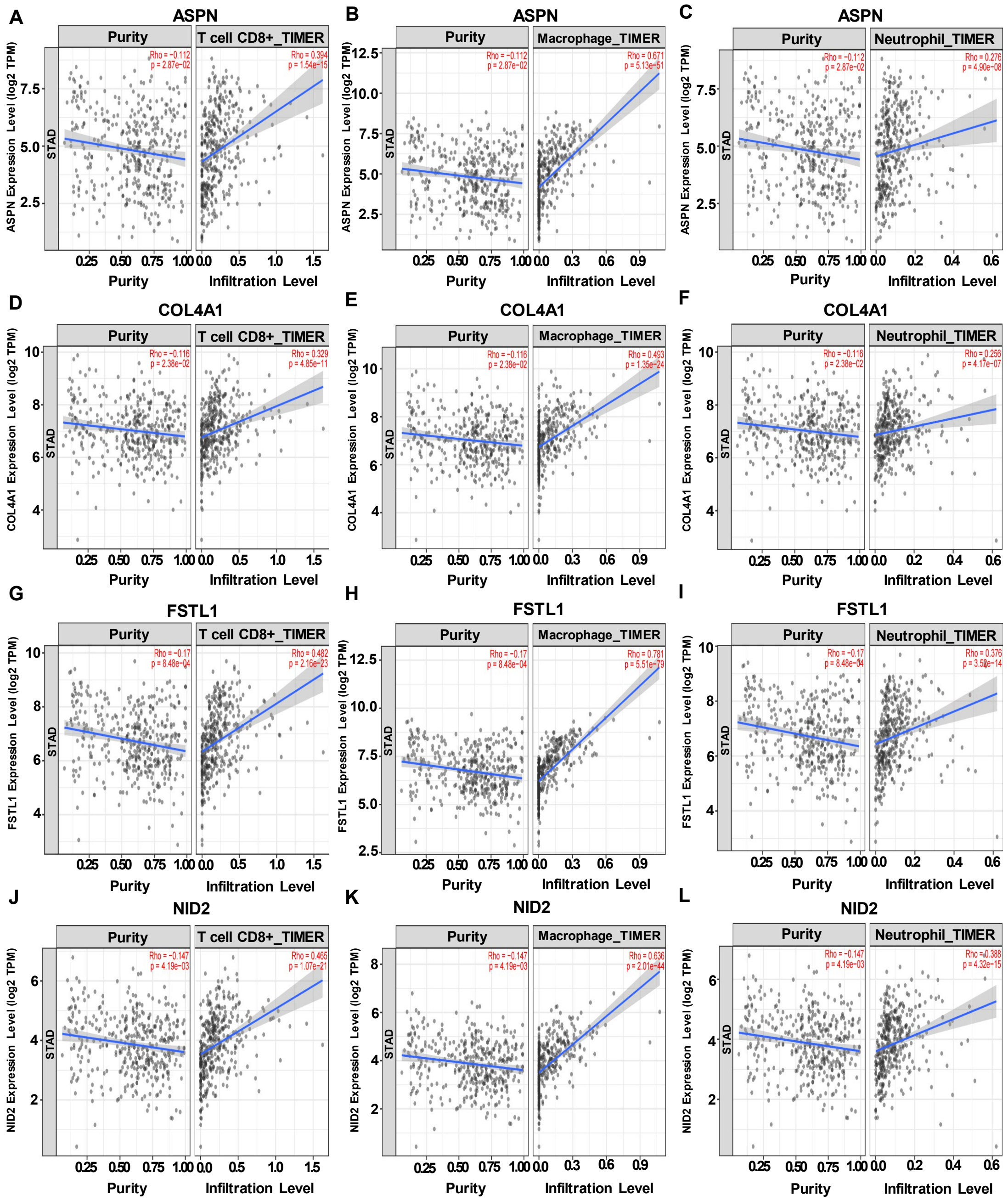

Figure S10: Immune cell-related expression of 4 prognosis-related genes.

(A-C) Expression of ASPN in three types of immune cells.

(D-F) Expression of COL4A1 in three types of immune cells.

(G-I) Expression of FSTL1 in three types of immune cells.

(J-L) Expression of NID2 in three types of immune cells.

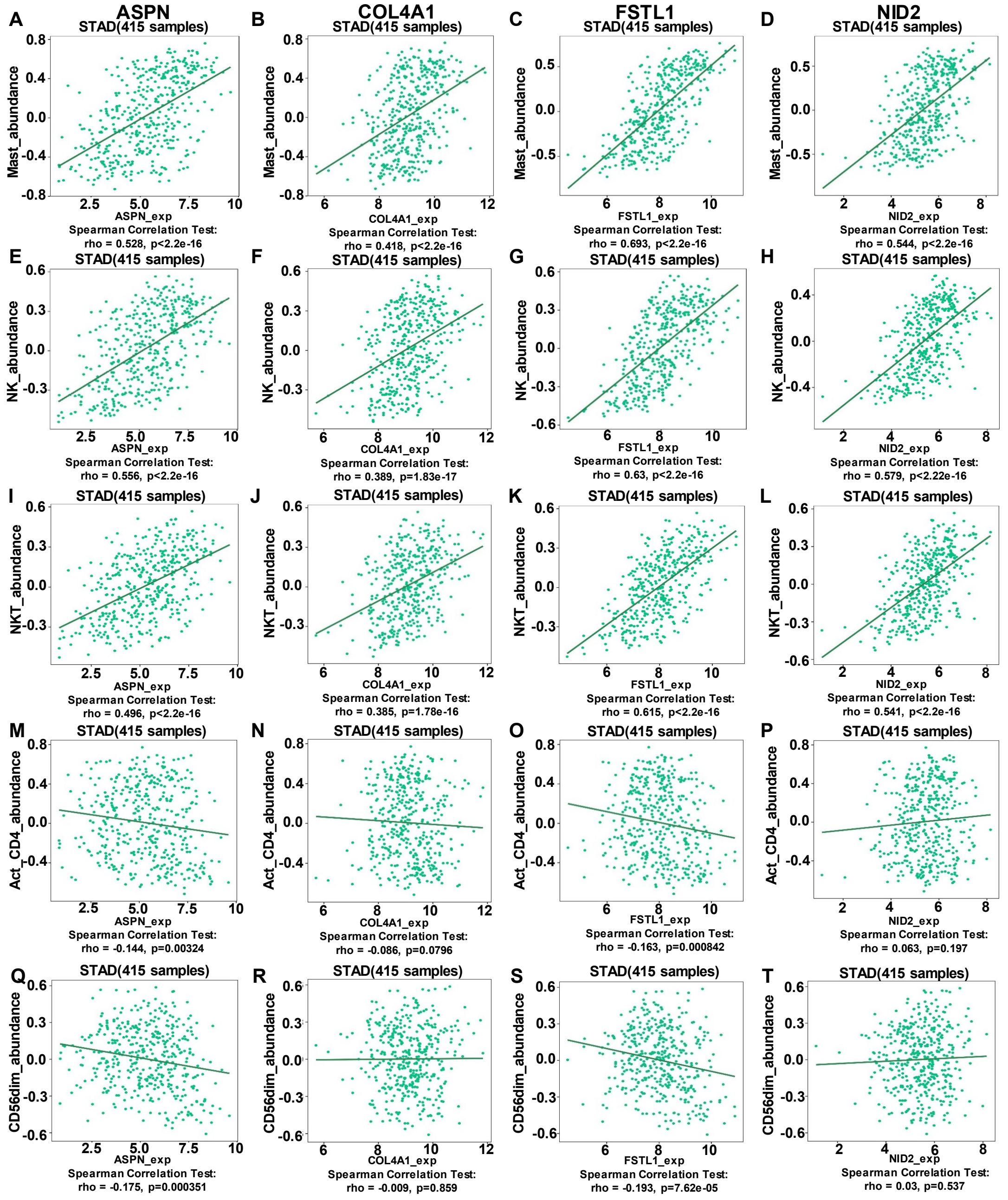

Figure S11: Lymphocyte-associated expression of 4 prognosis-related genes.

(A-D) Expression in mast cells (Mast).

(E-H) Expression in natural killer cells (NK).

(I-L) Expression in natural killer T cells (NKT).

(M-P) Expression in activated CD4 T cells (Act_CD4).

(Q-T) Expression in CD56dim natural killer cells (CD56dim).

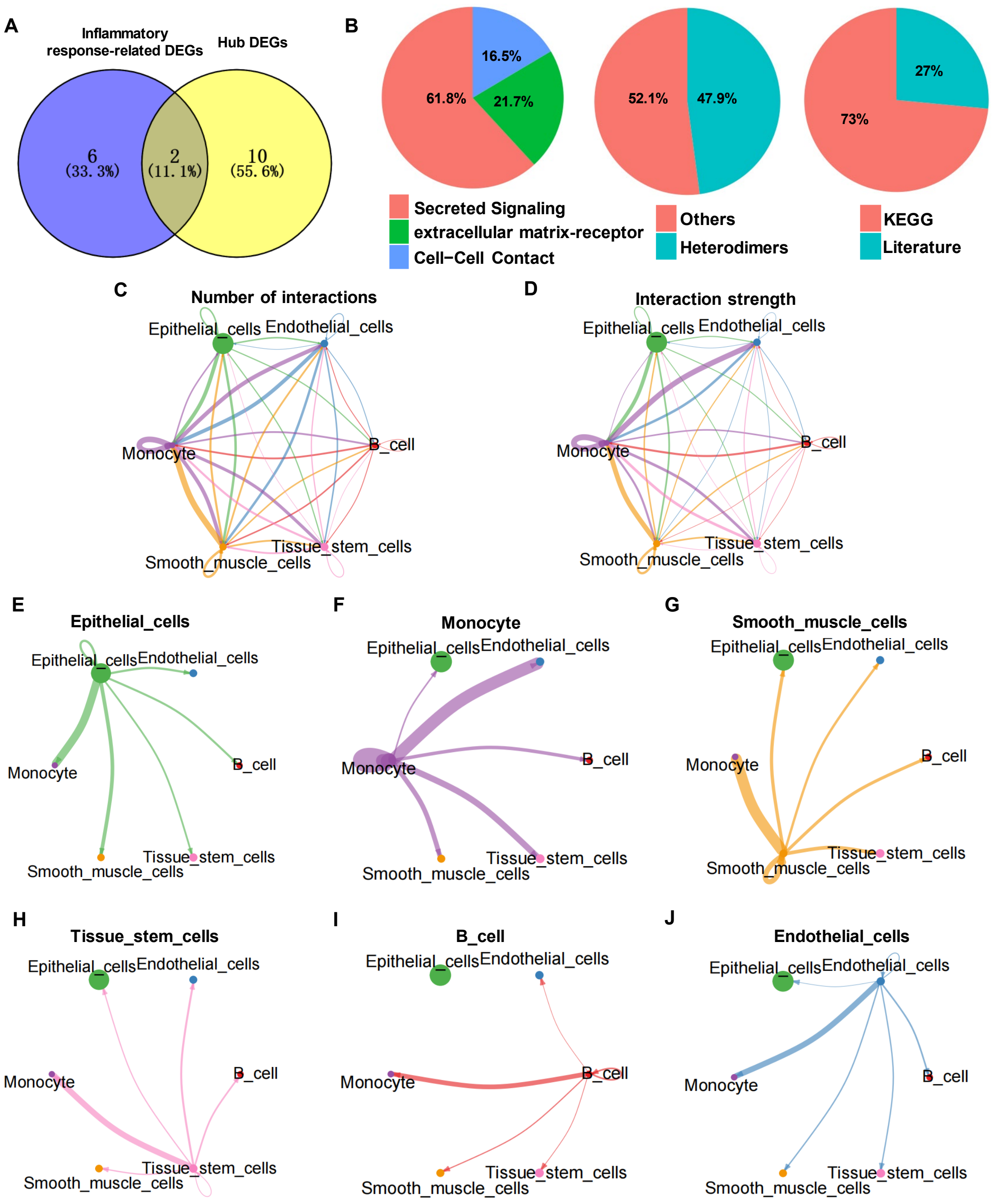

Figure S12: Cell–Cell communications between main six cell types.

(A) Venn diagram showing the intersection of 8 inflammatory response-related DEGs and 12 hub DEGs. **(B)** Composition type of ligand-receptor pair database.

(C) Network plot showing the interaction number. **(D)** Network plot showing the interaction strength. **(E-J)** Network plot showing the interaction of single cells. Sixteen types were annotated as epithelial cells, monocyte, smooth muscle cells, tissue stem cells, B cells, and endothelial cells.

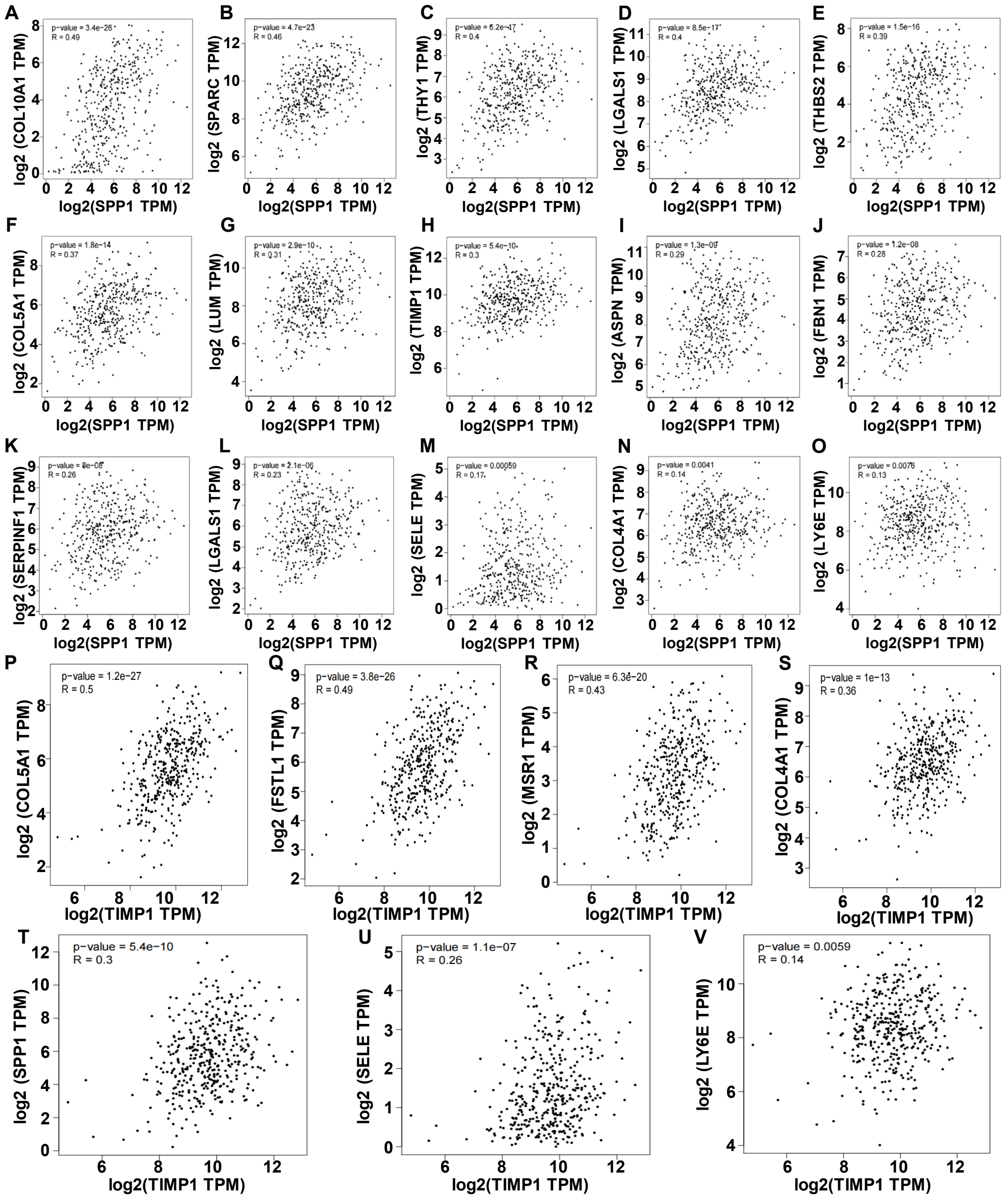

Figure S13: Correlation of SPP1 and TIMP1 with other gene expressions.

(A) SPP1-COL10A1. **(B)** SPP1-SPARC. **(C)** SPP1-THY1. **(D)** SPP1-LGALS1. **(E)** SPP1-THBS2. **(F)** SPP1-COL5A1. **(G)** SPP1-LUM. **(H)** SPP1-TIMP1. **(I)** SPP1-ASPN. **(J)** SPP1-FBN1. **(K)** SPP1-SERPINF1. **(L)** SPP1-LGALS1. **(M)** SPP1-SELE. **(N)** SPP1-COL4A1. **(O)** SPP1-LY6E. **(P)** TIMP1-COL5A1. **(Q)** TIMP1-FSTL1. **(R)** TIMP1-MSR1. **(S)** TIMP1-COL4A1. **(T)** TIMP1-SPP1. **(U)** TIMP1-SELE. **(V)** TIMP1-LY6E.
